# Systematic review and meta-analysis of lifestyle and reproductive factors associated with risk of breast cancer in Asian women

**DOI:** 10.1158/1055-9965.EPI-24-0005

**Published:** 2024-10-02

**Authors:** Boon Hong Ang, Soo-Hwang Teo, Weang-Kee Ho

**Affiliations:** 1 Cancer Research Malaysia, Level 1, Subang Jaya Medical Centre South Tower, No. 1, Jalan SS 12/1A, 47500 Subang Jaya, Selangor Darul Ehsan, Malaysia; 2 Faculty of Science and Engineering, School of Mathematical Sciences, University of Nottingham Malaysia, Jalan Broga , 43500 Semenyih, Selangor Darul Ehsan, Malaysia; 3 Faculty of Medicine, University Malaya Cancer Research Institute, University of Malaya, 50603 Kuala Lumpur, Wilayah Persekutuan Kuala Lumpur, Malaysia

**Keywords:** Breast cancer, Asian, risk factors, lifestyle, reproductive

## Abstract

**Introduction:**

Assessing breast cancer risks from lifestyle and reproductive factors is critical for developing population-specific risk prediction tools. However, limited studies have evaluated these risks in recent Asian birth cohorts.

**Methods:**

We systematically reviewed articles published from January 2010 to December 2023, examining breast cancer risk factors in Asian women. Data were described narratively, estimates pooled, and prevalence and attributable proportions compared across Asian populations.

**Results:**

Of the 128 studies reviewed, 103 reported adjusted effect sizes for meta-analysis. Lifestyle and reproductive factors were predictive of breast cancer risk in Asian women, with varying impacts on pre-menopausal and post-menopausal women. Relative risks were similar within Asian populations and in comparison to European populations, except for menarche, menopause, and hormone receptor therapy. However, risk factor distributions differed across populations. Whilst alcohol intake (21%) and oral contraceptive use (20%) emerged as the most attributable modifiable risk factors in Europeans, passive smoking (24%) and higher BMI (17%,≥24kg/m^2^ among post-menopausal women) were predominant in Asians.

**Conclusion:**

Our study shows that whilst the effects of lifestyle and reproductive breast cancer risk factors are largely similar across different populations, their distributions vary.

**Impact:**

Our analysis underscores the importance of considering population-specific risk factor distributions when developing risk prediction tools for Asian populations.

## Introduction

Breast cancer is the most prevalent cancer globally, but its incidence varies up to threefold across different regions (1). Whilst incidence rates have plateaued in some high-income countries with predominantly European populations since 2000, the incidence in Asia is projected to double between 2020 and 2040, largely due to changes in lifestyle and reproductive factors (1–5).

Some genetic factors associated with the risk of breast cancer have different prevalence in diverse populations, but the relative risks associated with these genetic factors are similar (6). For instance, protein-truncating germline variants in *BARD1 and CHEK2* are more common in women of European descent. However, the relative risks associated with these variants, as well as with protein-truncating variants in other rare cancer predisposition genes, are similar in European and Asian women (6,7). Lifestyle and reproductive factors associated with the risk of breast cancer also exhibit different distributions in diverse populations, but less is known about whether the relative risks associated with these factors are similar across different populations. One example of a possible difference is soy consumption, which has been shown to be associated with a lower risk of breast cancer in Asian women (8–11), whilst its effect in European women remains equivocal, likely due to varying intake levels influenced by lifestyle differences between the two populations (8,9,12).

Although a number of systematic reviews have explored the impact of lifestyle and reproductive factors on breast cancer risk in Asians, these have often focused on specific subsets of Asians (e.g., East Asians (13), West Asians (14), South East Asians (15), or South Asians (16)), specific risk factors (e.g., alcohol (17), induced abortion (18), or dietary factors (19,20)), or were conducted over a decade ago (21,22). Given the significant birth cohort effect, where women born in more recent cohorts have a significantly higher risk of breast cancer (23–25), we sought to evaluate the breast cancer risk associated with lifestyle and reproductive factors in an updated analysis of Asian populations. In this study, we undertook a systematic review and meta-analysis to summarise the evidence from studies published in the past 14 years that have quantified the impact of lifestyle and reproductive factors on breast cancer risk among Asian populations.

## Materials and Methods

### Search strategy and inclusion criteria

This systematic review adhered to the methodological standards outlined in the Preferred Reporting Items for Systematic Reviews and Meta-Analyses (PRISMA) guideline (26). A systematic literature search was conducted in PubMed and ProQuest to identify studies evaluating breast cancer risk among women in Asian countries, using the search terms “risk factor”, “breast cancer”, and “Asia” (Table S1). The reference lists of the identified studies were also reviewed to identify additional relevant studies. Studies were included in this review based on the following criteria: [1] prospective cohort, nested case-control, or case-control (sample size, n≥200) study designs; [2] reported effect sizes (Odds Ratios, OR; Risk Ratios/Relative Risks, RR) with 95% confidence intervals; and [3] inclusion of women of Asian descent. To account for potential birth cohort effects on breast cancer incidence (27), our search was limited to studies published from January 2010 to December 2023. The protocol for this review was not registered.

### Data extraction and quality assessment

Potentially relevant articles were initially screened based on titles and abstracts, followed by a full-text assessment for final eligibility. The inclusion of eligible studies was made through joint discussion and consultation with the review team. Data from each study were extracted using a standardised template, which included the following information: [1] population characteristics (such as region/country, sample size, birth cohort), [2] study design (prospective cohort, nested case-control, or case-control), [3] methodology (including data collection and analysis), and [4] study outcomes (factors associated with breast cancer risk and their relevant effect sizes). The quality of all included studies was assessed using the Newcastle-Ottawa Quality Assessment Scale (NOS), a validated scale for case-control and cohort studies (28). This assessment considered three categories: selection of participants (up to 4 points), comparability of study groups (up to 2 points), and outcome measurement (up to 3 points), resulting in an overall quality classification as good, fair, or poor.

### Factors associated with breast cancer risk

Predictive factors of breast cancer encompass various characteristics or exposures, including sociodemographic factors [e.g., age, education (level, years), employment (status, type), marital status (married, single, divorced, or widowed), and location (urban or rural)], personal and family history [e.g., history of benign breast disease (yes or no) and family history of breast cancer (yes or no)], reproductive factors [e.g., parity (status, number of children), age at first birth, breastfeeding (ever or never, duration), abortion (ever or never), age at menarche, menopausal status (pre- or post-menopause), and age at menopause], lifestyle factors [e.g., body mass index (BMI, kg/m^2^: overweight or obese), physical activity (active or inactive), smoking (ever or never), passive smoking (ever or never), sleeping pattern (regular or irregular, duration), alcohol intake (ever or never), green tea (more or less), vitamin D (more or less), isoflavone (more or less), and whole food (more or less) including meat, dairy, fish, fruits, vegetables, and soy], and exogenous hormonal factors [e.g., oral contraceptive use (OC: ever or never) and hormone receptor therapy (HRT: ever or never)]. The BMI classification for overweight and obese individuals used in this review adheres to two distinct sets of classifications by the World Health Organization: the standard BMI classification (29) (overweight=25-29kg/m^2^ and obese≥30kg/m^2^) and the Asian BMI classification (30), which includes slightly lower thresholds for Asian populations (overweight=23-24 kg/m^2^ and obese=27-29kg/m^2^).

## Data analysis

We described all the data narratively and estimated the associations of potential factors with breast cancer risk by pooling the results across studies using the DerSimonian and Laird random-effects model if heterogeneity p-value<0.05 (31) and Mantel-Haenszel method using fixed-effects model if heterogeneity p-value≥0.05 (32). Results were presented as odds ratios (OR) or risk ratios/relative risks (RR) with their corresponding 95% confidence intervals (CI), and forest plots were generated to illustrate the effect sizes of breast cancer risk factors by study design. Subgroup analyses were conducted based on menopausal status (pre-menopausal or post-menopausal), breast cancer subtype (luminal, human epidermal growth factor receptor 2 (HER2), or triple-negative breast cancer (TNBC)), and location (urban or rural) where data were available. For risk factors where only one study available, we included the reported effect sizes, and in cases where multiple effect sizes were reported for a risk factor, we meta-analysed the effect size generated using the highest category. Heterogeneity of pooled estimates across studies was assessed using Cochran’s Q and I^2^ statistics (33). Publication bias was evaluated using Egger’s test. A p-value<0.05 indicates the presence of a small study effect, whereby smaller studies have greater variations in their estimations and tend to be more scattered compared to larger studies. Additionally, we conducted a two-sample t-test to compare the effect sizes between study designs (Retrospective case-control versus Prospective cohort) and populations (Asian women versus European women). The attributable risk proportion (AR), quantifying the proportion of breast cancer incidence linked to a specific exposure or risk factor, was computed using Levin’s formula. It incorporates effect size and population-specific risk factor distribution [AR (%) = (((OR/RR - 1) * Prevalence) / (1 + ((OR/RR - 1) * Prevalence))) * 100] (34). Adjustments for country-specific distributions was made for Asian women where data were available, and attributable risk proportions were computed by country (including Singapore, South Korea, Taiwan, and Japan).

Data were analysed using Stata version 13.0 (Stata Corp., College Station., Texas, USA). All the reported p-values were two-sided and p-value<0.05 was considered statistically significant.

## Results

### Study characteristics

From 7,515 studies identified and screened, 128 studies published between 2010 and 2023, were included in qualitative synthesis (Figure S1). Overall, there were 95 retrospective case-control studies, 30 prospective cohort studies, and 3 nested case-control studies, involving 134,201 women diagnosed with breast cancer (Table S2-3) (35–162). Generally, prospective studies recruited larger samples (n=1,101-3,095,336) compared to retrospective studies (n=200-20,767). Prospective studies were primarily from high-income or middle-income countries such as Japan (n=17), South Korea (n=3), China (n=5), Taiwan (n=2), and Singapore (n=2), whilst retrospective studies were from a broader spectrum of countries, including high-income, middle-income, and low-income countries like China (n=19), Iran (n=19), India (n=12), Japan (n=10), Malaysia (n=7), Pakistan (n=4), South Korea (n=3), Vietnam (n=3), Saudi Arabia (n=3), Taiwan (n=2), Hong Kong (n=2), and Thailand (n=2). On average, prospective cohort studies had a significantly higher NOS score of 8 [standard deviation, sd=0.61], with more than three-quarters graded as good. By contrast, retrospective case-control studies had a lower NOS score of 6 [sd=1.08], with only one-third graded as good, whilst the remaining were of fair quality. More than three-quarters of the included studies analysed populations born after 1946.

### Factors associated with breast cancer risk in Asian women

**Figure 1:**
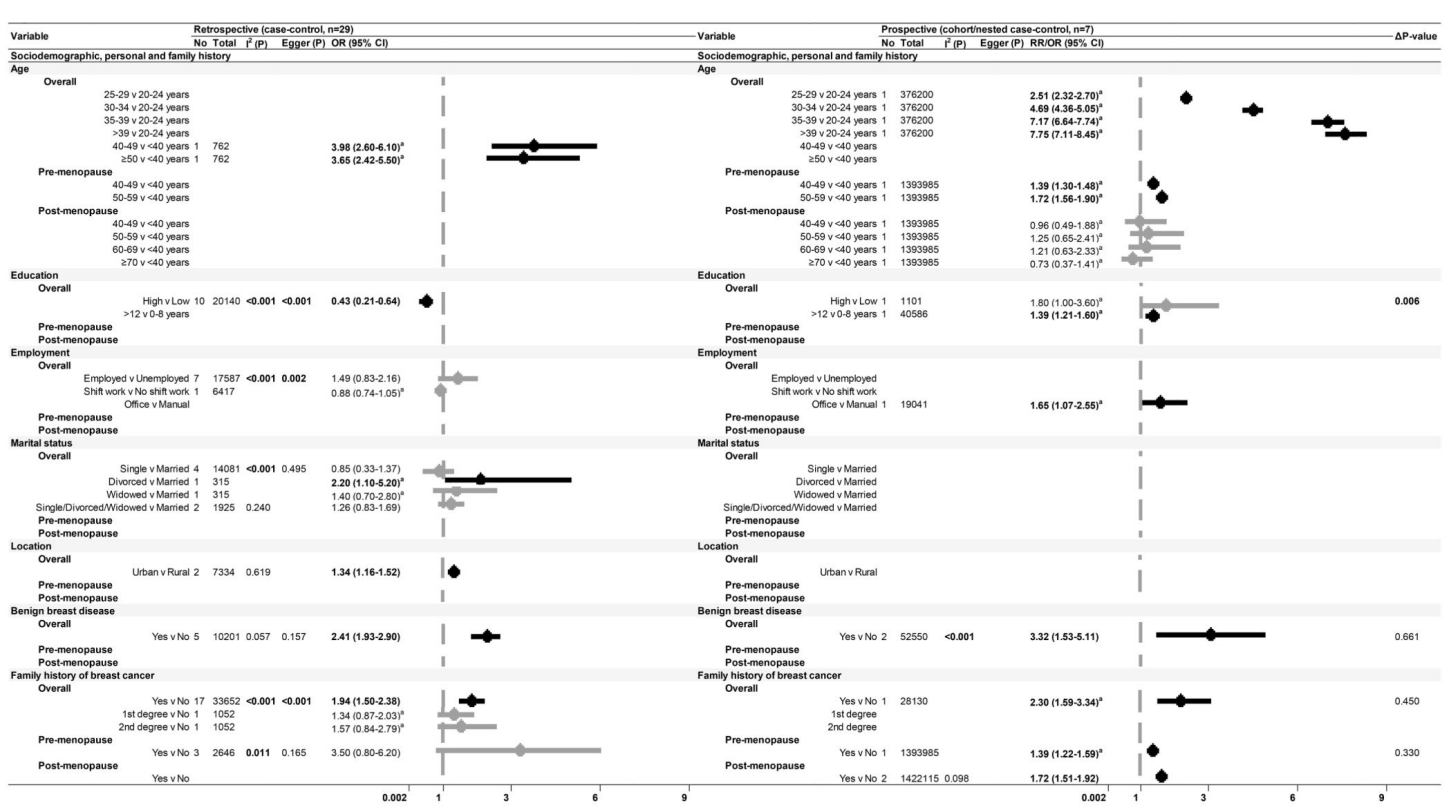
Forest plot of breast cancer risk and sociodemographic, personal and family history factors Meta-analysis representing the association of sociodemographic characteristics, personal history of benign breast disease, and family history of breast cancer with breast cancer risk, presented by study design in Asian populations for all women and stratified by menopausal status Abbreviations: No, Number of studies; Total, sample size; I ^2^(P), Heterogeneity test p-value; Egger (P), Egger test p-value; OR, Odds Ratio; RR, Risk Ratio/Relative Risk; 95% CI, 95% Confidence Interval. ^a^ Effect sizes as reported in the respective studies. Note: P<0.05 are presented in bold. **Δ**p-value presented in this table are referring to effect size comparison between study designs.

**Figure 2:**
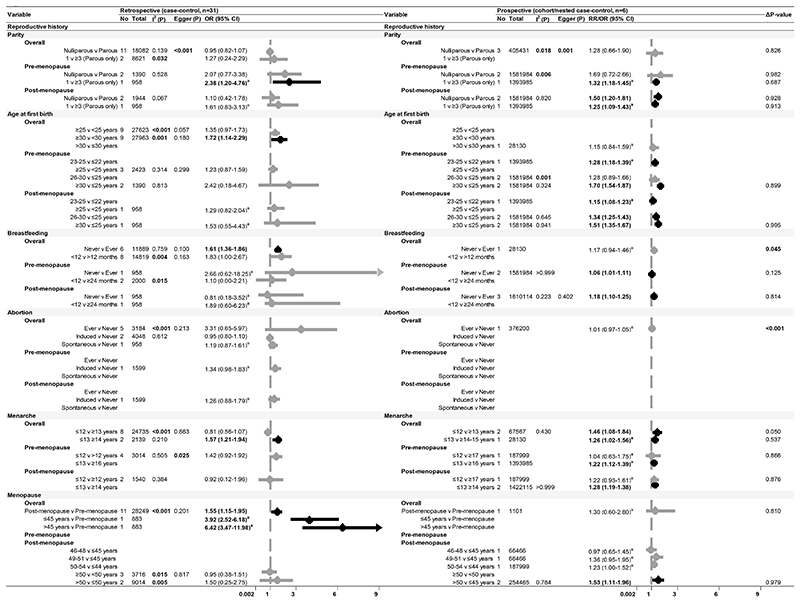
Forest plot of breast cancer risk and reproductive factors Meta-analysis representing the association of menstrual and reproductive history with breast cancer risk, presented by study design in Asian populations for all women and stratified by menopausal status Abbreviations: No, Number of studies; Total, sample size; I^2^(P), Heterogeneity test p-value; Egger (P), Egger test p-value; OR, Odds Ratio; RR, Risk Ratio/Relative Risk; 95% CI, 95% Confidence Interval. ^a^ Effect sizes as reported in the respective studies. Note: P<0.05 are presented in bold. **Δ**p-value presented in this table are referring to effect size comparison between study designs.

**Figure 3:**
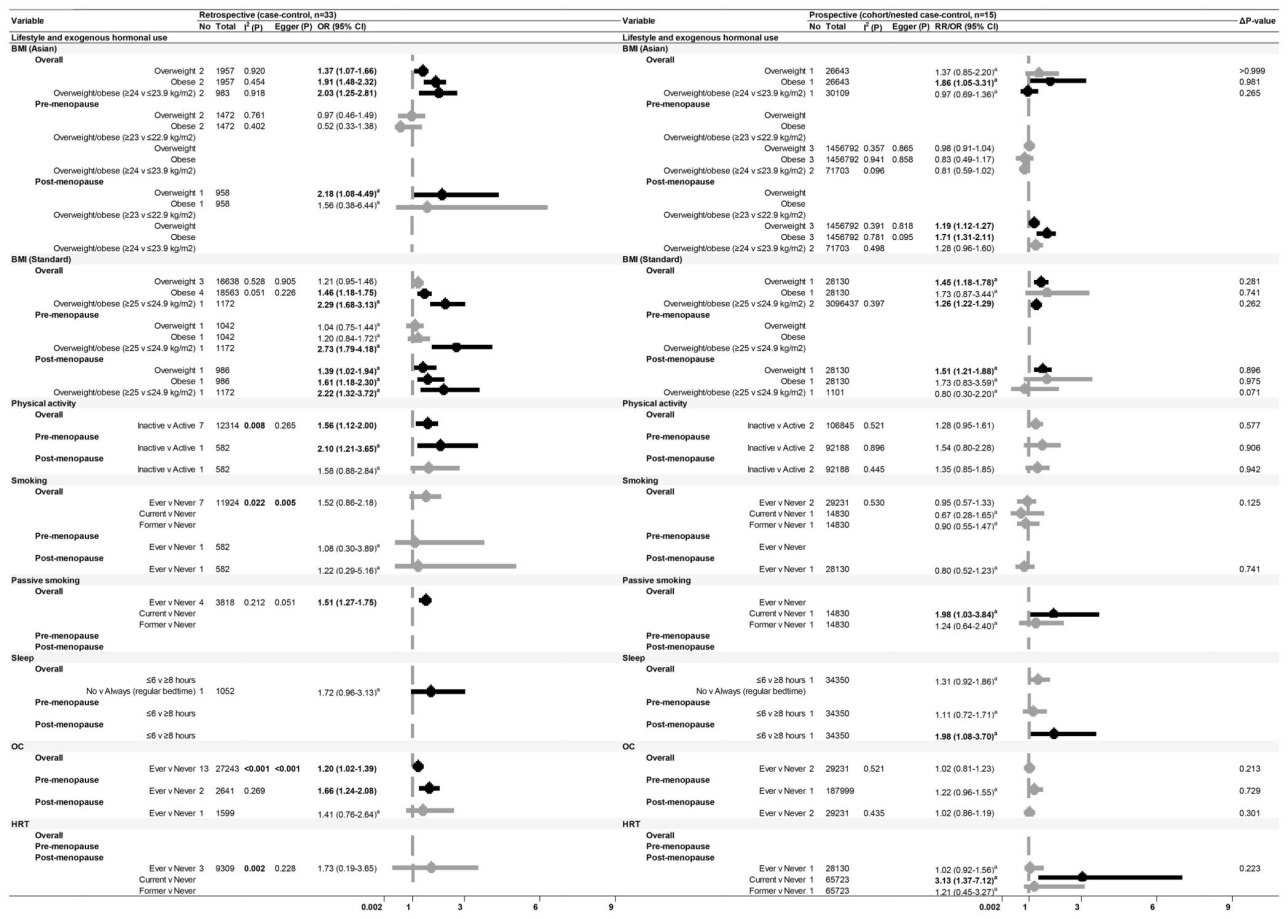
Forest plot of breast cancer risk and lifestyle factors (excluding diet) Meta-analysis representing the association of modifiable lifestyle and exogenous hormonal use with breast cancer risk, presented by study design in Asian populations for all women and stratified by menopausal status Abbreviations: No, Number of studies; Total, sample size; I^2^(P), Heterogeneity test p-value; Egger (P), Egger test p-value; OR, Odds Ratio; RR, Risk Ratio/Relative Risk; 95% CI, 95% Confidence Interval; BMI, Body mass index; BMI Asian, Asian BMI classification; BMI Standard, European BMI classification; OC, Oral contraceptives; HRT, Hormone receptor therapy. ^a^ Effect sizes as reported in the respective studies. Note: P<0.05 are presented in bold. **Δ**p-value presented in this table are referring to effect size comparison between study designs.

**Figure 4:**
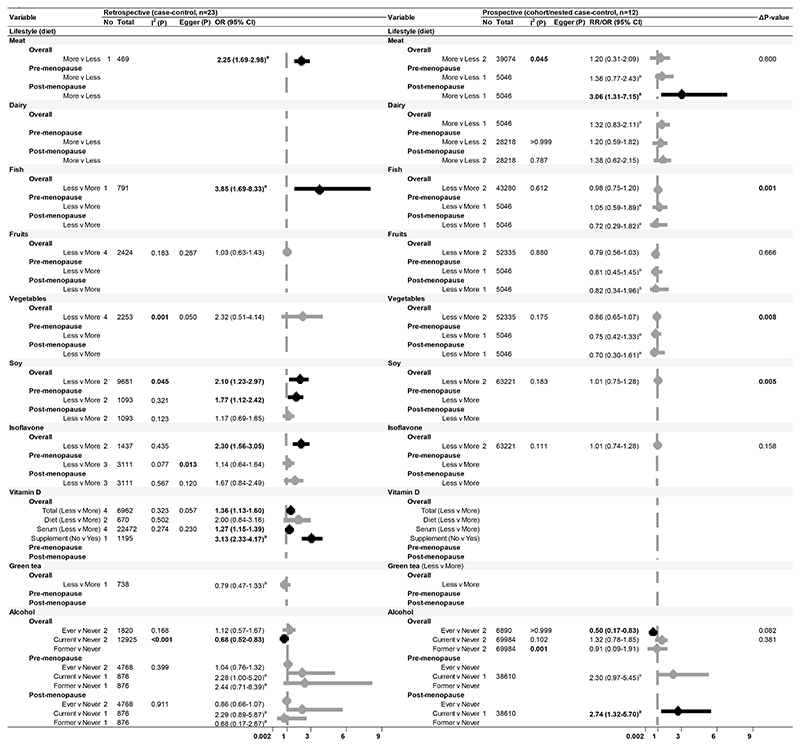
Forest plot of breast cancer risk and lifestyle factors (diet) Meta-analysis representing the association of modifiable dietary factors with breast cancer risk, presented by study design in Asian populations for all women and stratified by menopausal status Abbreviations: No, Number of studies; Total, sample size; I^2^(P), Heterogeneity test p-value; Egger (P), Egger test p-value; OR, Odds Ratio; RR, Risk Ratio/Relative Risk; 95% CI, 95% Confidence Interval. ^a^ Effect sizes as reported in the respective studies. Note: P<0.05 are presented in bold. **Δ**p-value presented in this table are referring to effect size comparison between study designs

After excluding 25 studies (see Figure S1 for reasons of exclusion), 103 studies (35–40,42–45,49–52,54–58,60–75,77–91,93,94,96,98–101,103–106,108–112,115,117,118,120,121,123,125,128–131,133–137,140–146,148–157,159–162), including 32 prospective studies that conducted multivariable adjustment, were included in the meta-analysis (Figure 1-4). The NOS scores of these studies ranged from 4 to 9 and were graded either as good or fair quality, with none graded as poor (Table S2-3). Overall, the pooled effect sizes of significant risk factors did not differ between retrospective and prospective studies. Publication bias was evident in retrospective studies for level of education, use of OC, and family history of breast cancer (Egger’s test p-value<0.001). Smaller sample studies (n<1000) reported significantly higher estimates with wider confidence intervals.

### Sociodemographic, personal and family history

We identified older age (25 years onwards), employment (office worker), marital status (divorced), and urban residence as significant sociodemographic factors associated with a higher risk of breast cancer in Asian women (Figure 1). Longer years of education were associated with an increased risk of breast cancer in a prospective study, whilst a lower level of education was identified as a risk factor in retrospective settings. Non-modifiable factors, such as a history of benign breast disease and family history of breast cancer, were also associated with an increased risk. Specifically, family history had a significant impact on the luminal breast cancer subtype (Table S4).

The impact of age was notable among pre-menopausal women, with elevated risks observed between ages 40-49 (RR 1.39, 95% CI: 1.30-1.48) and 50-59 (RR 1.72, 95% CI: 1.56-1.90). Conversely, family history of breast cancer demonstrated significant relevance among post-menopausal women compared to their pre-menopausal counterparts [RR (95% CI): 1.97 (1.16-2.79) versus 1.39 (1.22-1.59), Figure 1].

### Reproductive history

Having fewer children (only one child), delayed parity (>30 years), early menarche (<14 years), and delayed menopause (>50 years) were associated with an increased risk of breast cancer (Figure 2). Notably, never breastfeeding and post-menopausal status were predictive of risk in retrospective settings only. These factors, apart from the number of children and age at menopause, demonstrated significant effects, particularly on the luminal breast cancer subtype (Table S4). Whilst induced abortion was associated with a higher risk of luminal breast cancer (OR 1.27, 95% CI: 1.04-1.50), spontaneous abortion was protective against the risk of luminal breast cancer (OR 0.60, 95% CI: 0.31-0.90).

Never breastfeeding showed a more pronounced effect among post-menopausal women compared to pre-menopausal women [RR (95% CI: 1.18 (1.10-1.25) versus 1.06 (1.01-1.11)], and nulliparity was significant only in the former (RR 1.50, 95% CI: 1.20-1.81, Figure 2). In contrast, delayed parity had a more pronounced impact on pre-menopausal women compared to post-menopausal women, particularly between the ages of 23 and 25 years [RR (95% CI): 1.28 (1.18-1.39) versus RR (95% CI): 1.15 (1.08-1.23)] and 30 years onwards [RR (95% CI): 1.70 (1.54-1.87) versus RR (95% CI): 1.51 (1.35-1.67)]. The risk of breast cancer was higher among rural-living women with fewer children [OR 2.38, 95% CI: 1.33-4.17, Table S5].

### Lifestyle and exogenous hormonal use

Among modifiable factors, increased BMI (overweight or obese), passive smoking (current exposure), irregular sleeping pattern, and HRT use (current user), were positively associated with breast cancer risk (Figure 3). Physical inactivity and OC use were significant risk factors observed in retrospective settings only. Smoking, on the other hand, showed no significant association with breast cancer risk. Additionally, higher BMI was linked to an increased risk of developing luminal and HER2 breast cancer subtypes (Table S4).

Overweight and obese post-menopausal women exhibited significant relative risks of 1.19 (95% CI: 1.12-1.27) and 1.71 (95% CI: 1.31-2.11), respectively, but these factors were not associated with risks in pre-menopausal women [RR (95% CI): 0.98 (0.91-1.04) and 0.83 (0.49-1.17), respectively, Figure 3]. Similarly, a shorter duration of sleep (6 hours or less) was associated with a higher risk of breast cancer among post-menopausal women only (RR 1.98, 95% CI: 1.08-3.70). Both physical inactivity and OC use were predictive of risk among pre-menopausal women but lacked prospective evidence. We also identified several other risk factors that were not meta-analysed in this review due to limited evidence, including hair colouring, betel nut or tobacco chewing, and stress.

### Lifestyle (diet)

We conducted a meta-analysis on dietary factors, comparing the lowest and highest categories reported in respective studies (Figure 4). In retrospective settings, lower consumption of fish, soy, and isoflavones was associated with a higher breast cancer risk, whilst increased meat consumption was linked to elevated risk. Vitamin D, particularly through supplements, and alcohol intake, showed a protective effect. Conversely, consumption of fruits, vegetables, dairy, and green tea did not predict breast cancer risk.

The impact of meat consumption and alcohol intake was significant among post-menopausal women only [RR (95% CI): 3.06 (1.31-7.15) and 2.74 (1.32-5.70), respectively, Figure 4]. Conversely, soy intake was predictive of risk among pre-menopausal women (OR 1.77, 95% CI: 1.12-2.42) but lacked prospective evidence.

Heterogeneity among prospective studies included in the meta-analysis was mostly absent, except for the history of benign breast disease (p-value<0.001, Figure 1). This variability was attributed to study design, notably a nested case-control study, which reported a higher relative risk compared to a cohort study [OR (95% CI): 4.30 (3.20-5.20) versus RR (95% CI): 2.47 (2.26-2.71)].

### Comparison of breast cancer risk factors across populations

The distributions of risk factors varied within Asian countries, particularly when comparing East and South East Asians, as depicted in Table 1. Specifically, there were notable variations in the distributions of OC use (38% versus 9-17%) and age at menarche (35% versus 20-28%). Consequently, we computed attributable risk proportions separately for each Asian country where data were available. To ascertain the similarity of cancer risk associated with lifestyle and reproductive factors between Asian and European populations, we compared pooled effect sizes from the present study with those from published meta-analyses of prospective studies in women of European descent (Table 1, S6-7 for comparisons, Table S8 for citations). Whilst most of the reported relative risks were largely comparable between the two populations, notable differences were observed.

### Effect sizes of breast cancer risk factors

In general, the risks associated with age at menarche were higher in Asian women compared to European women [<13 years, RR (95% CI): 1.46 (1.08-1.84) versus 1.07 (1.05-1.09), **Δ**p-value<0.001, Table 1]. Conversely, smoking (current smoker) and diet (fruit consumption) were significantly higher in the latter [RR (95% CI): 1.24 (1.07-1.42) and 1.09 (1.02-1.16), respectively] but not significant in the former. Whilst alcohol intake posed a risk for European women, it exhibited a protective effect among Asian women [RR (95% CI): 0.50 (0.17-0.83) versus 1.46 (1.34-1.58), **Δ**p-value=0.001]. In post-menopausal women, the risks in Asian women were higher than that in European women for overweight BMI [Standard BMI classification, RR (95% CI): 1.51 (1.21-1.88) versus 1.12 (1.06-1.18), **Δ**p-value=0.008], obese BMI [Standard BMI classification, RR (95% CI): 1.73 (0.83-3.59) versus 1.16 (1.08-1.25), **Δ**p-value=0.022], family history of breast cancer [RR (95% CI): 1.72 (1.51-1.92) versus 0.96 (0.82-1.12), **Δ**p-value=0.015], and age at menopause [>50 years, RR (95% CI): 1.53 (1.11-1.96) versus 1.12 (1.07-1.17), **Δ** p-value=0.016, Table S7]. The relative risk for current HRT users was higher in Asians [RR (95% CI): 3.13 (1.37-7.12) versus 1.08 (1.04-1.12), **Δ**p-value<0.001], whilst for former HRT users, it was higher in Europeans [RR (95% CI): 1.68 (1.64-1.72) versus 1.21 (0.45-3.27), **Δ**p-value=0.015].

### Distributions of breast cancer risk factors

Whilst some risk factors showed marginal discrepancies (within ±10%) between Asian and European women, the majority exhibited differences, with higher proportions observed in European populations (Table 1, S6-7). European women reported higher proportions of nulliparity, delayed parity, early menarche, delayed menopause, higher BMI, physically inactivity, exogenous hormonal usage, alcohol consumption, smoking, and a history of abortion or family history of breast cancer, with variations exceeding 10%. Conversely, the prevalence of passive smoking was higher in Asian women compared to European women (current exposure: 32% versus 11% and former exposure: 50% versus 32%).

### Attributable risk proportions of breast cancer risk factors

We computed the attributable risk proportions of breast cancer risk factors in both populations using data from the literature to determine preventable proportion of breast cancer cases (Table 1, S6-7). The primary risk factor contributing to breast cancer in Asian women was passive smoking, with a population attributable risk of 24% (95% CI: 23-24) for current exposure. Conversely, family history of breast cancer was the most significant risk factor for European women, with an attributable risk of 32% (95% CI: 32-32). Among modifiable risk factors, whilst OC use (AR 20%, 95% CI: 20-20) and alcohol intake (AR 21%, 95% CI: 21-21) were the most significant attributable risk factors in women of European descent, passive smoking remains the major contributor to breast cancer risk among Asian women. Delayed parity (≥30 years, AR 9%, 95% CI: 9-9) and physical inactivity (AR 15%, 95% CI: 15-15) emerged as the most significant attributable risk factors among pre-menopausal women for Asian and European women, respectively (Table S6). HRT use was prevalent among post-menopausal women, with different patterns observed between Asian and European populations (Table S7). For current users, the attributable risk was significant in Asian women (AR 22%, 95% CI: 22-22), whereas for former users, it was significant in European women (AR 19%, 95% CI: 19-19). Additionally, shorter sleep duration (AR 22%, 95% CI: 22-22) and higher BMI [AR (95% CI): overweight, 5% (5–5) and obese, 12% (12–12)] were significant attributable risk factors among Asian post-menopausal women.

History of benign breast disease and age at menarche accounted for attributable risks of 11% (95% CI: 11-11) and 8% (95% CI: 8-8), respectively (Table 1). Other risk factors such as family history, age at menopause, nulliparity, and breastfeeding contributed 5% or less to the population risk of breast cancer (Table 1, S6-7). Notably, abortion, menopausal status, physical activity, smoking, and OC use were not considered due to either non-significant associations or conflicting findings, particularly regarding alcohol intake. Attributable risk was not computed for most dietary factors, due to limitation in prevalence data, where the lowest and highest categories were similar in proportions.

## Discussion

This systematic review and meta-analysis represent the first comprehensive analysis of breast cancer risk factors in recent birth cohorts among Asian populations. Lifestyle factors (including BMI, passive smoking, sleeping pattern, exogenous hormonal use, physical activity, meat consumption, and alcohol intake) and reproductive factors (including parity, menarche, menopause, and breastfeeding) emerged as predictive of breast cancer risk in Asian women, with their impacts varying based on menopausal status. Although relative risks remained similar within Asians and compared to Europeans, risk factor distributions varied across populations. As a result, alcohol intake (21%) and OC use (20%) were identified as the most significant modifiable risk factors among European women, whilst passive smoking (24%) and higher BMI (17% in post-menopausal women) were predominant among Asian women.

We found that the relative risks of significant risk factors were largely similar between Asian and European populations, with exceptions noted for age at menarche, age at menopause, and HRT for current users, where the relative risks were higher in Asian women, particularly in more recent birth cohorts (72,121,146,162–164). Asian prospective studies that investigated the impact of these factors on breast cancer risk, including women born after 1946, reported higher relative risks compared to the corresponding European studies, which primarily recruited women born before 1946. The reasons for this difference in more recent cohorts warrant further research.

We observed that the relative risks for overweight and obese BMI were higher among post-menopausal Asian women when standard BMI thresholds were applied to both Asian and European women. This leads to an overestimation of risk for overweight Asian women (RR 1.51, 95% CI: 1.21-188) and an underestimation of risk for obese Asian women (RR 1.73, 95% CI: 0.83-3.59). However, when the Asian BMI thresholds were used for Asians and standard BMI thresholds were used for Europeans, there were no differences in the effect sizes. Specifically, for overweight women, the relative risk was 1.19 (95% CI: 1.12-1.27) compared to 1.12 (95% CI: 1.06-1.18), with a **Δ**p-value of 0.267. For obese women, the relative risk was 1.71 (95% CI: 1.31-2.11) compared to 1.16 (95% CI: 1.08-1.25), with a **Δ**p-value of 0.087 (Table S7). It is known that metabolic risks are greater in Asians than Europeans at a given BMI, and thus, BMI thresholds for defining overweight and obese should be lower for Asians (30).

The protective effect of alcohol intake among Asians could be due to its lower prevalence compared to Europeans (16.5% versus 58%) (74,162), and there is evidence suggesting that moderate consumption of alcohol can be beneficial (165). We found that the effect sizes and directions of associations for smoking were different in Asian and European women, but the effects were not statistically significant in Asians. This might be because of insufficient sample size to detect small effects, as only one prospective study evaluated smoking by type of smokers (current and former) (84). On the other hand, the effect sizes and directions of associations for physical inactivity and OC use were similar in Asian and European women, but the effects were not statistically significant in Asians. Likewise, this could be attributed to insufficient sample size, as only three prospective studies evaluated physical activity (74,81,144) and OC use (121,123,146) each. Alternatively, it could be due to lower prevalence in Asians compared to Europeans, particularly for OC use (17.4-38.3% versus 82%) (69,123,146,166). Despite the widespread belief in the protective effects of dietary factors, whilst we did observe some associations for increased meat consumption and alcohol intake among post-menopausal Asian women based on one study each, our findings suggest no association between dietary factors (such as dairy, fish, vegetables, soy, and isoflavone) and breast cancer risk in Asian and European populations, aligning with recent reviews (20,167,168). Induced abortion was not associated with breast cancer risk in either the Asian or European population in prospective settings (160,169). Moreover, relying on self-reported data for sensitive information may suffer from reporting bias (170).

The impact of family cancer history, reproductive history, and BMI on breast cancer risk varied by menopausal status among Asian women, unlike what was observed among European women. Specifically, the relative risks for family cancer history, delayed parity, and breastfeeding status were comparable between pre- and post-menopausal European women (171–174). The association of BMI by menopausal status also differs between the populations. Whilst a higher BMI was protective against pre-menopausal breast cancer risk in European (167) and Asian women, it was not statistically significant in the latter, consistent with the report from the World Cancer Research Fund/American Institute for Cancer Research (WCRF/AIRC) and a systematic review (167,175,176). It was proposed that the observed protective effect could be attributed to hormonal changes, particularly in progesterone, which differ between obese and non-obese women. Obese young women may experience a more pronounced decline in progesterone levels towards the end of their menstrual cycle, occurring later compared to non-obese women (176).

Understanding the proportion of population attributable risk provides valuable insights into the aetiology of breast cancer and can guide targeted prevention efforts aimed at reducing the overall disease burden in the population. Whilst 21% of breast cancer cases in Europeans could be prevented by reducing alcohol intake and a further 20% through avoiding OC use, our study reveals that up to 24% of breast cancer cases in Asians are attributed to exposure to second-hand smoke, with an additional 17% of risk can be reduced by avoiding overweight and obesity among post-menopausal women. Although the point estimates for physical activity were similar in Europeans and Asians, the lack of a statistically significant effect in Asians precludes our ability to draw conclusions about the attributable risk of this risk factor in Asians.

This review has several limitations. One key limitation is the relatively small number of Asian prospective studies published in the last 14 years (n=33), specifically exploring lifestyle and reproductive breast cancer risk factors. Of these, 32 studies that contributed to the meta-analysis were of East and South East Asian ethnic subgroups, potentially limiting the generalisability of the findings to other Asian populations, particularly those in South, West, and Central regions. Additionally, subgroup analyses based on menopausal status, breast cancer subtype, and location, as well as the computation of attributable risk proportions by Asian countries, were limited by the availability of published data, including effect sizes and prevalence of risk factors.

In summary, although we observed similar relative risks, there were notable differences in the distributions of risk factors between Asian and European populations. This suggests that by adjusting for population-specific risk factor distributions, risk prediction tools derived from European studies could potentially be adapted for predicting breast cancer risk in Asian populations. Our findings underscore the importance of using population-specific BMI classification: to accurately assess the risk associated with overweight and obese BMI in Asians, it is recommended to use Asian-specific BMI thresholds as approved by World Health Organization (30), whilst also considering menopausal status for risk estimation. Lastly, incorporating variables such as passive smoking, which holds significance among Asians, has the potential to enhance the precision of risk prediction for Asian populations.

The evolving exposure patterns to specific risk factors in Asian women are expected to further elevate breast cancer incidence in this region, emphasising the importance of accurate risk estimations for the development of risk prediction tools. Our analysis of Asian women, alongside comparisons with studies of European women, highlights the need of tailoring risk prediction tools based on population-specific BMI classification and the distributions of risk factors among recent birth cohorts.

## Supplementary Material

Sup Fig 1, Sup Tab1-8

## Figures and Tables

**Table 1:** Effect sizes, distributions, and attributable proportions of breast cancer risk factors in prospective studies among Asian and European populations

Variable	OR/RR (95% CI)	Asian								OR/RR (95% CI)	European		P-value
		Singapore		South Korea		Taiwan		Japan			%	AR^c^ (95% CI)	
		%	AR^b^ (95% CI)	%	AR^b^ (95% CI)	%	AR^b^ (95% CI)	%	AR^b^ (95% CI)				
**Overall**													
**Personal and family history**													
Benign breast disease (Yes v No)	**3.32 (1.53-5.11)**	5.2	**11 (11-11)**	3.7	**8 (8-8)**					**2.07 (1.64-2.61)**	6.4	**6 (6-6)**	0.105
Family history of breast cancer (Yes v No)	**2.30 (1.59-3.34)^a^**	2.5	**3 (3-3)**	3.4	**4 (4-4)**	3.3	**4 (4-4)**	1.4	**2 (2-2)**	**3.90 (2.03-7.49)**	16.4	**32 (32-32)**	0.631
**Reproductive history**													
Parity (Nulliparous v Parous)	1.28 (0.66-1.90)	7.2	2 (2-2)	6.7	2 (2-2)	4.2	1 (1-1)	8.8	2 (2-2)	**1.15 (1.03-1.28)**	15.8	**2 (2-2)**	0.695
Age at first birth (>30 v ≤30 years)	1.15 (0.84-1.59)^a^	8.6	1 (1-1)										
Breastfeeding (Never v Ever)	1.17 (0.94-1.46)^a^	30.4	5 (5-5)							1.08 (0.97-1.20)	37.0	3 (3-3)	0.489
Abortion (Yes v No)	1.01 (0.97-1.05)^a^					3.1	0 (0-0)			0.97 (0.84-1.13)^a^	26.8		0.681
Menarche													
<13 v ≥14 years	**1.46 (1.08-1.84)**			5.5	**2 (2-2)**			13.3	**6 (6-6)**	**1.07 (1.05-1.09)**	36.2	**2 (2-2)**	**<0.001**
<14 v ≥14 years	**1.26 (1.02-1.56)^a^**	34.9	**8 (8-8)**	19.7	**5 (5-5)**			27.8	**7 (7-7)**				
Menopause (Post- v Pre-menopause)	1.30 (0.60-2.80)^a^			28.4	8 (8-8)								
**Lifestyle and exogenous hormonal use**													
BMI (Asian, ≥24 v ≤23.9 kg/m^2^)													
Overweight	1.37 (0.85-2.20)^a^							29.7	10 (10-10)				
Obese	**1.86 (1.05-3.31)^a^**							3.2	**3 (3-3)**				
BMI (Standard, ≥25 v ≤24.9 kg/m^2^)													
Overweight	**1.45 (1.18-1.78)^a^**	33.3	**13 (13-13)**										
Obese	1.73 (0.87-3.44)^a^	10.1	7 (7-7)										
Physical activity (Inactive v Active)	1.28 (0.95-1.61)							36.6	9 (9-9)	**1.15 (1.03-1.28)**	53.5	**7 (7-7)**	0.664
Smoking													
Current v Never	0.67 (0.28-1.65)^a^							13.0		**1.24 (1.07-1.42)**	8.2	**2 (2-2)**	**0.023**
Former v Never	0.90 (0.55-1.47)^a^							4.6		**1.13 (1.06-1.21)**	35.6	**4 (4-4)**	0.082
Passive smoking													
Current n Never	**1.98 (1.03-3.84)^a^**							31.5	**24 (23-24)**	1.02 (0.89-1.16)	11.0	0 (0-0)	0.273
Former v Never	1.24 (0.54-2.40)^a^							50.0	11 (11-11)	1.00 (0.91-1.10)	32.0	0 (0-0)	0.659
Sleep (≤6hours v ≥8 hours)	1.31 (0.92-1.86)^a^							29.3	8 (8-8)				
OC (Ever v Never)	1.02 (0.81-1.23)	38.3	1 (1-1)	17.4	0 (0-0)			18.0	0 (0-0)	**1.30 (1.13-1.49)**	82.0	**20 (20-20)**	0.403
**Lifestyle (diet)**													
Alcohol (Ever v Never)	**0.50 (0.17-0.83)**							16.5		**1.46 (1.34-1.58)**	58.0	**21 (21-21)**	**0.001**
Meat (More v Less)	1.20 (0.31-2.09)									1.08 (0.98-1.19)			0.587
Dairy (More v Less)	1.32 (0.83-2.11)^a^									0.89 (0.81-0.98)			0.498
Fish (Less vs More)	0.98 (0.75-1.20)									0.99 (0.85-1.15)			0.964
Fruits (Less v More)	0.79 (0.56-1.03)									**1.09 (1.02-1.16)**			**0.002**
Vegetables (Less v More)	0.86 (0.65-1.07)									1.01 (0.94-1.09)			0.107
Soy (Less v More)	1.01 (0.75-1.28)												
Isoflavone (Less v More)	1.01 (0.74-1.28)									1.00 (0.70-1.30)^a^			0.978

Abbreviations: OR, Odds Ratio; RR, Risk Ratio/Relative Risk; 95% CI, Confidence Interval; Population, Population distribution; AR, Attributable risk; BMI, Body mass index; BMI Asian, Asian BMI classification; BMI Standard, European BMI classification; OC, Oral contraceptives; HRT, Hormone receptor therapy.

a Effect sizes as reported in the respective studies.

b AR values for Asians were determined using pooled effect sizes and risk factor distributions from each Asian country, with 95% confidence intervals determined using the delta method.

c AR values for Europeans were determined using effect sizes and risk factor distributions extracted from cohort studies, systematic reviews, meta-analyses, or collaborative projects, with 95% confidence intervals determined using the delta method.

Note: P<0.05 are presented in bold. Sociodemographic factors are not included due to limited published effect sizes in the West for comparison. **Δ**p-value presented in this table are referring to effect size comparisons between populations. The AR values in the table indicate that when stated as X% of a risk factor, it represents the percentage of breast cancer cases attributed to that specific risk factor.

## Data Availability

All data generated or analysed during this study are included in this published article [and its supplemental information files].
