# Supplementary material for: Systematic review and meta-analysis of lifestyle and reproductive factors associated with risk of breast cancer in Asian women
Source: Cancer Epidemiol Biomarkers Prev. Author manuscript; Available in PMC 2025 Feb 25. (PMC7617425; doi:10.1158/1055-9965.EPI-24-0005)
Supplement: Sup Fig 1, Sup Tab1-8 [file EMS203365-supplement-Sup_Fig_1__Sup_Tab1_8.pdf]

SUPPLEMENTAL FIGURE LEGEND

Figure S1: Flowchart on study selection using PRISMA

SUPPLEMENTAL MATERIALS

Table S1: Keyword combinations used for online database systematic search

No.	Keywords
**1**	"Socio*demograph*" OR Demograph* OR Lifestyle* OR Menstrua* OR Reproduct* OR "Non*genetic" OR "Modifiable factor*" OR "Risk factor*" OR Factor* OR Prognostic
**2**	Age OR Gender OR Race OR Ethnicity OR Education OR Income OR "Marital status" OR Occupation OR Employment OR "Household size" OR "Household composition" OR Geograph* OR Anthropometr* OR Weight OR BMI OR "Body Mass Index" OR "Physical activity" OR Exercise OR Sedentar* OR Nutrition OR Diet* OR Smok* OR Alcohol OR Caffeine OR Coffee OR "Psychological stress" OR Stress OR "Sleep cycle" OR Sleep OR Menarche OR Pregnancy OR Menopause OR "Pre*menopaus*" OR "Post*menopaus*" OR Parity OR Breastfeeding OR Menstrua* OR Miscarriage* OR Abortion* OR "Oral contraceptive" OR "Hormone replacement therapy" OR HRT OR Children OR "Family history"
**3**	"Breast cancer" OR "Breast carcinoma"
**4**	Asia OR Asian* OR Afghanistan OR Armenia OR Azerbaijan OR Bahrain OR Bangladesh OR Bhutan OR Brunei OR Cambodia OR China OR Cyprus OR Georgia OR India OR Indonesia OR Iran OR Iraq OR Israel OR Japan OR Jordan OR Kazakhstan OR Kuwait OR Kyrgyzstan OR Laos OR Lebanon OR Malaysia OR Maldives OR Mongolia OR Myanmar OR Nepal OR "North Korea" OR Oman OR Pakistan OR Palestine OR Philippines OR Qatar OR Russia OR "Saudi Arabia" OR Singapore OR "South Korea" OR "Sri Lanka" OR Syria OR Taiwan OR Tajikistan OR Thailand OR Timor-Leste OR Turkey OR Turkmenistan OR "United Arab Emirates" OR Uzbekistan OR Vietnam OR Yemen
**5**	**#1 AND #2 AND #3 AND #4**

Figure S1: Flowchart on study selection using PRISMA

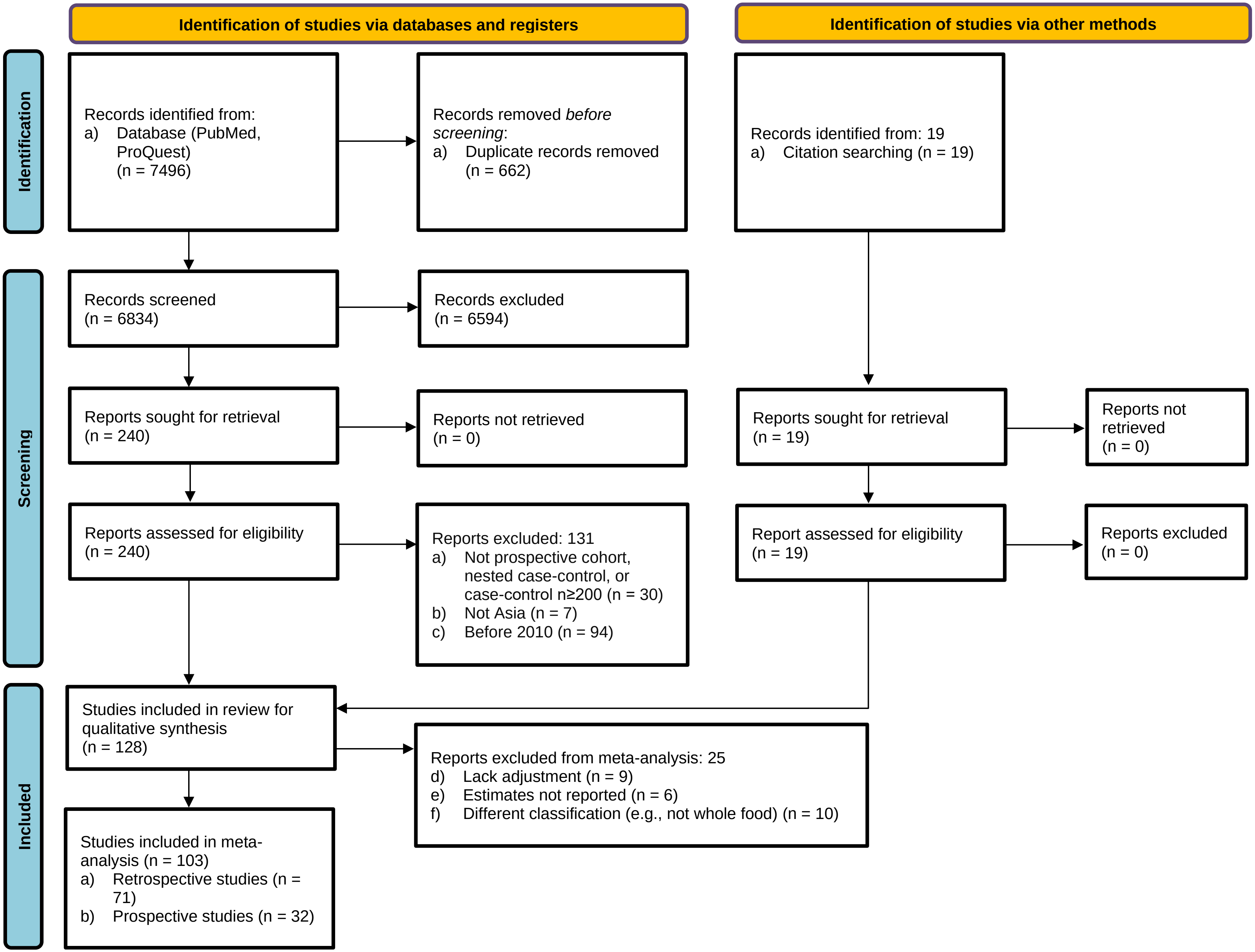

Table S2: Characteristics of included studies

Region	Country	Retrospective (case-control) *Sample size, n (range): 200 - 20767*				Prospective (cohort/nested case-control) *Sample size, n (range): 1101 - 1393985*			
		**No. of study**	**Reference**	**NOS[a]** *mean (sd)*	**Cohort[b]** *≥1946*	**No. of study**	**Reference**	**NOS[a]** *mean (sd)*	**Cohort[b]** *<1946*
**Overall**		95	-	6 (1.08)	98%	33	-	8 (0.61)	64%
**East Asia**	China	19	(1-36)	6 (1.23)	94%	5	(21,32,37-65)	8 (0.56)	62%
	Japan	10				17			
	Taiwan	4				4			
	South Korea	3				3			
	Hong Kong	2				-			
**West Asia**	Israel	1	(66-93)	6 (0.63)	100%	1	(94)	9 (0)	100%
	Armenia	1				-			
	Iran	19				-			
	Saudi Arabia	3				-			
	Turkey	1				-			
	Yemen	1				-			
	Jordan	1				-			
	Palestine	1				-			
**Southeast Asia**	Indonesia	1	(95-107)	6 (1.14)	100%	-	(108-110)	7 (0.58)	67%
	Malaysia	7				-			
	Thailand	2				-			
	Vietnam	3				-			
	Philippines	-				1			
	Singapore	-				2			
**South Asia**	India	12	(111-126)	6 (1.06)	100%	-	-	-	-
	Pakistan	4							
**Central Asia**	Kazakhstan	1	(127,128)	5 (1.41)	100%	-	-	-	-
	Afghanistan	1							

Abbreviations: NOS, Newcastle-Ottawa Scale; sd, standard deviation.
[a] *Study quality (NOS): 0-3: Poor; 4-7: Fair; 6-9: Good.*
[b] *Birth cohort: <1946 (Silent generation); ≥1946 (Baby boomer or X generation).*

Table S3: Summary of individual study characteristics

First author_Year	Region	Country	Design	Sample	Case	Total	Cohort*	NOS
Akbari_2020	WA	Iran	Case-control	SC	732	1316	≥1946	5
Al Qadire_2018	WA	Jordan	Case-control	MC	405	823	≥1946	5
Alsolami_2019	WA	Saudi Arabia	Case-control	SC	214	432	≥1946	6
Bano_2016	SA	Pakistan	Case-control	PB	1238	2246	≥1946	6
Baset_2021	CA	Afghanistan	Case-control	SC	201	402	≥1946	6
Bashamakha_2019	WA	Yemen	Case-control	SC	105	315	≥1946	6
Bhadoria_2013	SA	India	Case-control	SC	320	640	≥1946	6
Bui_2022	SEA	Vietnam	Case-control	MC	490	958	≥1946	7
Butler_2010	SEA	Singapore	Cohort	PB	629	34028	<1946	8
Cao_2019	EA	Japan	Cohort	PB	236	34350	<1946	7
Chang_2017	EA	Taiwan	Case-control	SC	233	469	≥1946	5
Chaveepojnkamjorn_2017	SEA	Thailand	Case-control	SC	257	514	≥1946	7
Chen_2016	EA	Taiwan	Cohort	PB	14008	1393985	≥1946	8
Cho_2010	EA	South Korea	Case-control	SC	358	718	≥1946	5
Chuang_2015	EA	Taiwan	Nested case-control	PB	4884	24420	<1946	8
Das_2012	SA	India	Case-control	SC	105	210	≥1946	4
Dianatinasab_2017	WA	Iran	Case-control	SC	526	1052	≥1946	6
Ekpanyaskul_2010	SEA	Thailand	Case-control	SC	516	1032	≥1946	5
El Sharif_2021	WA	Palestine	Case-control	SC	237	474	≥1946	6
Elkum_2014	WA	Saudi Arabia	Case-control	SC	534	1172	≥1946	6
Fu_2015	EA	China	Case-control	SC	372	791	≥1946	5
Gao_2013	EA	China	Case-control	PB	669	1351	≥1946	7
Gathani_2017	SA	India	Case-control	MC	2101	4356	≥1946	7
Ghiasvand_2012	WA	Iran	Case-control	SC	493	986	≥1946	6
Ghiasvand_2011	WA	Iran	Case-control	SC	521	1042	≥1946	6
Gibson_2010	SEA	Philippines	Nested case-control	PB	123	1101	≥1946	7
Goldberg_2015	WA	Israel	Cohort	PB	2073	40586	<1946	9
Guo_2014	EA	China	Cohort	PB	229	26643	≥1946	8
Hajian-Tilaki_2011	WA	Iran	Case-control	MC	100	300	≥1946	6
Hajian-Tilaki_2011	WA	Iran	Case-control	MC	100	300	≥1946	5
Hajian-Tilaki_2012	WA	Iran	Case-control	MC	100	300	≥1946	5
Haseen_2015	SA	Pakistan	Case-control	SC	175	350	≥1946	5
Heidari_2018	WA	Iran	Case-control	SC	134	401	≥1946	6
Ho_2020	SEA	Singapore	Cohort	PB	474	28130	<1946	7
Huang_2023	EA	Taiwan	Case-control	SC	297	582	≥1946	6
Islam_2013	EA	Japan	Case-control	SC	1754	5262	≥1946	6
Islam_2012	EA	Japan	Case-control	SC	706	2118	≥1946	6
Itoh_2014	EA	Japan	Case-control	MC	405	810	≥1946	6
Iwasaki_2014	EA	Japan	Case-control	MC	369	738	≥1946	6
Jamshidinaeini_2016	WA	Iran	Case-control	SC	135	270	≥1946	6
Joukar_2016	WA	Iran	Case-control	SC	225	450	≥1946	5
Jung_2016	EA	South Korea	Cohort	PB	989	66466	≥1946	7
Kawai_2013	EA	Japan	Case-control	SC	1092	4252	<1946	6
Kawai_2011	EA	Japan	Cohort	PB	241	19227	<1946	8
Kawase_2010	EA	Japan	Case-control	SC	1803	5409	≥1946	7
Keshet-Sitton_2017	WA	Israel	Case-control	MC	110	252	≥1946	5
Khachatryan_2011	WA	Armenia	Case-control	SC	150	302	≥1946	7
Kim_2017	EA	South Korea	Cohort	PB	72	5046	≥1946	8
Kiyabu_2015	EA	Japan	Cohort	PB	556	38234	<1946	8
Kojima_2017	EA	Japan	Cohort	PB	119	23172	<1946	8
Lai_2011	EA	Taiwan	Cohort	PB	780	65723	≥1946	8

First author_Year	Region	Country	Design	Sample	Case	Total	Cohort*	NOS
Lee_2011	EA	China	Case-control	SC	200	400	≥1946	5
Li_2022	EA	China	Case-control	SC	495	973	≥1946	6
Li_2017	EA	China	Case-control	SC	1256	2672	≥1946	6
Li_2015	EA	China	Nested case-control	PB	1709	6489	<1946	7
Liu_2017	EA	China	Case-control	MC	1489	2978	≥1946	7
Liu_2011	EA	China	Case-control	PB	669	1351	≥1946	7
Liu_2016	EA	China	Cohort	PB	5094	68253	<1946	8
Lodha_2011	SA	India	Case-control	PB	215	430	≥1946	7
Lodha_2011	SA	India	Case-control	PB	215	430	≥1946	7
Lu_2017	EA	China	Case-control	PB	818	1753	≥1946	6
Maleki_2020	WA	Iran	Case-control	SC	958	1925	≥1946	6
Marzbani_2019	WA	Iran	Case-control	SC	212	620	≥1946	6
Matalqah_2011	SEA	Malaysia	Case-control	SC	150	300	≥1946	5
Mizoo_2013	EA	Japan	Case-control	PB	472	936	≥1946	6
Mohite_2015	SA	India	Case-control	MC	217	434	≥1946	5
Nagata_2013	EA	Japan	Case-control	SC	153	584	≥1946	6
Nagrani_2016	SA	India	Case-control	SC	1633	3137	≥1946	6
Nagrani_2016	SA	India	Case-control	SC	1637	3152	≥1946	5
Naz_2015	WA	Iran	Case-control	SC	108	216	≥1946	6
Nguyen_2016	SEA	Vietnam	Case-control	SC	294	588	≥1946	5
Nitta_2016	EA	Japan	Cohort	PB	273	38610	<1946	7
Park_2021	EA	South Korea	Cohort	PB	23584	3095336	≥1946	9
Park_2015	EA	South Korea	Case-control	MC	3634	20767	≥1946	7
Rajbongshi_2015	SA	India	Case-control	SC	100	200	≥1946	5
Razif_2011	SEA	Malaysia	Case-control	MC	216	432	≥1946	7
Safabakhsh_2020	WA	Iran	Case-control	SC	150	300	≥1946	7
Safabakhsh_2022	WA	Iran	Case-control	SC	150	300	≥1946	7
Sari_2020	EA	Japan	Cohort	PB	138	19041	<1946	7
Sepandi_2014	WA	Iran	Case-control	SC	197	11850	≥1946	5
Sezer_2011	WA	Turkey	Case-control	SC	172	555	≥1946	6
Shahar_2010	SEA	Malaysia	Case-control	MC	70	208	≥1946	5
Shahril_2013	SEA	Malaysia	Case-control	PB	382	764	≥1946	8
Shamsi_2020	SA	Pakistan	Case-control	MC	411	1195	≥1946	7
Shamsi_2013	SA	Pakistan	Case-control	MC	297	883	≥1946	6
Shen_2023	EA	China	Cohort	PB	11875	376200	≥1946	8
Shetty_2021	SA	India	Case-control	SC	120	240	≥1946	5
Shin_2016	EA	Japan	Cohort	PB	718	49552	<1946	8
Shirabe_2021	EA	Japan	Cohort	PB	825	47614	<1946	7
Shridhar_2018	SA	India	Case-control	PB	400	754	≥1946	8
Su_2022	EA	China	Cohort	PB	3674	53267	≥1946	8
Sulaiman_2011	SEA	Malaysia	Case-control	PB	382	764	≥1946	7
Suzuki_2013	EA	Japan	Cohort	PB	452	47289	<1946	7
Suzuki_2010	EA	Japan	Cohort	PB	572	50757	<1946	7
Suzuki_2011	EA	Japan	Cohort	PB	452	41594	<1946	7
Suzuki_2011	EA	Japan	Cohort	PB	652	53578	<1946	7
Suzuki_2013	EA	Japan	Cohort	PB	234	36164	<1946	7
Suzuki_2017	EA	Japan	Cohort	SC	325	30109	≥1946	7
Tajaddini_2015	WA	Iran	Case-control	SC	306	615	≥1946	5
Takeuchi_2021	EA	Japan	Cohort	PB	2329	187999	<1946	8
Takizawa_2018	EA	Japan	Case-control	SC	1256	4186	<1946	6
Tan_2018	SEA	Malaysia	Case-control	MC	3683	7663	≥1946	5
Tehranian_2010	WA	Iran	Case-control	SC	312	624	≥1946	5
Toi_2013	EA	Japan	Case-control	PB	306	968	≥1946	9

First author_Year	Region	Country	Design	Sample	Case	Total	Cohort*	NOS
Toleutay_2013	CA	Kazakhstan	Case-control	SC	114	310	≥1946	4
Tong_2014	EA	China	Case-control	SC	312	624	≥1946	5
Trieu_2017	SEA	Vietnam	Case-control	MC	269	788	≥1946	5
Tse_2015	EA	Hong Kong	Case-control	MC	747	1528	≥1946	9
Veisy_2015	WA	Iran	Case-control	MC	235	470	≥1946	6
Vishwakarma_2022	SA	India	Case-control	SC	187	474	≥1946	6
Wada_2015	EA	Japan	Cohort	PB	172	15607	≥1946	7
Wada_2013	EA	Japan	Cohort	PB	166	14830	<1946	8
Wahidin_2018	SEA	Indonesia	Case-control	MC	381	762	≥1946	5
Wang_2016	EA	Hong Kong	Case-control	MC	918	1841	≥1946	7
Wang_2020	EA	China	Case-control	SC	3792	7974	≥1946	6
Wang_2019	EA	China	Case-control	PB	328	984	≥1946	7
Wang_2013	EA	China	Case-control	PB	123	492	≥1946	7
Wang_2022	EA	Taiwan	Cohort	PB	112	5879	≥1946	8
Wu_2014	EA	China	Case-control	PB	1517	3090	≥1946	9
Xing_2010	EA	China	Case-control	SC	1417	3004	≥1946	5
Xu_2020	EA	China	Case-control	SC	51	314	≥1946	6
Yen_2016	SEA	Malaysia	Case-control	SC	122	243	≥1946	5
Yousef_2013	WA	Saudi Arabia	Case-control	SC	120	240	≥1946	6
Yu_2010	EA	South Korea	Case-control	SC	358	718	≥1946	5
Yuan_2019	EA	China	Case-control	MC	794	1599	≥1946	6
Zhang_2010	EA	China	Case-control	MC	438	876	≥1946	9
Zhang_2011	EA	China	Case-control	MC	1009	2018	≥1946	9
Zhu_2011	EA	China	Case-control	MC	183	375	≥1946	6

Abbreviations: EA, East Asia; WA, West Asia; SA, South Asia; SEA, South East Asia; CA, Central Asia; PB, Population based; MC, Multi centre; SC, Single centre; NOS, Newcastle-Ottawa Quality Assessment Scale.
Birth cohort: <1946 - Silent generation; ≥1946 - Baby boomer or X generation.

Table S4: Breast cancer risk factors by breast cancer subtype in Asian populations

Variable	Subgroup	No	Total	I^2 (P)	Reference	OR/RR (95% CI)
**Age**						
**40-49 v <40 years**	Luminal	1	2672		(34)	0.72 (0.50-1.06)[a]
	HER2	1	2672			0.85 (0.67-1.07)[a]
	TNBC	1	2672			0.53 (0.15-1.91)[a]
**≥60 v <40 years**	Luminal	1	2672			1.41 (0.62-3.24)[a]
	HER2	1	2672			0.67 (0.38-1.19)[a]
	TNBC	1	2672			1.27 (0.86-1.87)[a]
**Family history of breast cancer**						
**Yes v No**	Luminal A	1	3004		(24)	**2.24 (1.35-3.72)[a]**
	Luminal B	1	3004			2.15 (1.00-4.63)[a]
	HER2	1	3004			1.64 (0.62-4.37)[a]
	TNBC	1	3004			1.53 (0.75-3.15)[a]
**Menarche**						
**≤12 v >12 years**	Luminal A	2	3962	0.711	(24,107)	**2.42 (1.30-3.54)**
	Luminal B	2	3962	0.633		1.33 (0.38-2.27)
	HER2	2	3962	0.862		2.07 (0.48-3.65)
	TNBC	2	3962	0.660		1.62 (0.55-2.70)
**≤13 v >13 years**	Luminal	1	2672			1.28 (0.99-1.67)[a]
	HER2	1	2672			0.75 (0.29-1.95)[a]
	TNBC	1	2672			1.22 (0.81-1.83)[a]
**≤13 v >13 years (≤40 years)**	Luminal A	1	7974			1.10 (0.64-1.85)[a]
	Luminal B	1	7974			0.97 (0.74-1.27)[a]
	HER2	1	7974			1.08 (0.61-1.89)[a]
	TNBC	1	7974			0.93 (0.61-1.39)[a]
**≤13 v >13 years (>40 years)**	Luminal A	1	7974			1.01 (0.85-1.18)[a]
	Luminal B	1	7974			0.99 (0.59-1.15)[a]
	HER2	1	7974			1.27 (0.97-1.67)[a]
	TNBC	1	7974			1.04 (0.80-1.37)[a]
**Parity**						
**Nulliparous v Parous**	Luminal A	1	3004		(20,24)	0.61 (0.28-1.35)[a]
	Luminal B	1	3004			0.66 (0.24-1.82)[a]
	HER2	1	3004			0.48 (0.14-1.69)[a]
	TNBC	1	3004			0.33 (0.11-1.01)[a]
**Nulliparous v Parous (≤40 years)**	Luminal A	1	7974			**2.50 (1.14-5.56)[a]**
	Luminal B	1	7974			**2.50 (1.61-4.00)[a]**
	HER2	1	7974			1.32 (0.47-3.70)[a]
	TNBC	1	7974			1.18 (0.55-2.50)[a]
**Nulliparous v Parous (>40 years)**	Luminal A	1	7974			**2.08 (1.37-3.23)[a]**
	Luminal B	1	7974			**1.75 (1.18-2.63)[a]**
	HER2	1	7974			1.30 (0.58-2.86)[a]
	TNBC	1	7974			1.82 (0.55-2.50)[a]
**Age at first birth**						
**≥25-29 v <25 years**	Luminal	1	2672		(20,24,34)	**1.41 (1.17-1.70)[a]**
	Luminal A	1	3004			0.96 (0.78-1.18)[a]
	Luminal B	1	3004			0.81 (0.58-1.13)[a]
	HER2	2	5676	0.129		1.11 (0.73-1.49)
	TNBC	2	5676	0.123		1.11 (0.89-1.32)
**≥25-29 v <25 years (≤40 years)**	Luminal A	1	7974			0.65 (0.40-1.05)[a]
	Luminal B	1	7974			0.88 (0.68-1.14)[a]
	HER2	1	7974			0.99 (0.59-1.67)[a]
	TNBC	1	7974			0.92 (0.62-1.36)[a]
**≥25-29 v <25 years (>40 years)**	Luminal A	1	7974			**1.24 (1.07-1.44)[a]**
	Luminal B	1	7974			0.92 (0.81-1.06)[a]
	HER2	1	7974			0.88 (0.69-1.12)[a]
	TNBC	1	7974			1.02 (0.80-1.30)[a]
**≥30 v <25 years**	Luminal	1	2672			1.19 (0.75-1.90)[a]
	Luminal A	1	3004			0.96 (0.67-1.40)[a]
	Luminal B	1	3004			0.51 (0.24-1.10)[a]
	HER2	2	5676	>0.999		0.86 (0.13-1.59)
	TNBC	2	5676	0.699		0.78 (0.39-1.17)
**≥30 v <25 years (≤40 years)**	Luminal A	1	7974			0.48 (0.18-1.27)[a]
	Luminal B	1	7974			0.78 (0.50-1.22)[a]
	HER2	1	7974			1.05 (0.44-2.54)[a]
	TNBC	1	7974			0.86 (0.43-1.73)[a]
**≥30 v <25 years (>40 years)**	Luminal A	1	7974			**1.58 (1.21-2.07)[a]**
	Luminal B	1	7974			1.09 (0.84-1.42)[a]
	HER2	1	7974			0.77 (0.45-1.32)[a]
	TNBC	1	7974			0.79 (0.45-1.37)[a]
**Menopause**						
**Post-menopause v Pre-menopause**	Luminal	1	2672		(24,34)	**1.32 (1.05-1.65)[a]**

Variable		Subgroup	No	Total	I^2 (P)	Reference	OR/RR (95% CI)
		Luminal A	1	3004			**0.72 (0.55-0.94)[a]**
		Luminal B	1	3004			**0.64 (0.41-0.99)[a]**
		HER2	2	5676	0.709		0.92 (0.54-1.30)
		TNBC	2	5676	0.762		0.93 (0.70-1.15)
	**≥55 v ≤44 years**	Luminal A	1	3004			1.76 (0.83-3.75)[a]
		Luminal B	1	3004			0.64 (0.21-1.90)[a]
		HER2	1	3004			0.72 (0.24-2.20)[a]
		TNBC	1	3004			2.24 (0.77-6.54)[a]
**Breastfeeding**							
	**Ever v Never**	Luminal	1	2672		(20,24,34)	0.92 (0.76-1.32)[a]
		Luminal A	1	3004			**2.27 (1.59-3.23)[a]**
		Luminal B	1	3004			**1.85 (1.05-3.23)[a]**
		HER2	2	5676	0.298		2.29 (0.91-3.66)
		TNBC	2	5676	0.198		1.55 (0.93-2.17)
	**Ever v Never (≤40 years)**	Luminal A	1	7974			1.33 (0.59-3.03)[a]
		Luminal B	1	7974			**1.72 (1.14-2.63)[a]**
		HER2	1	7974			1.25 (0.52-3.03)[a]
		TNBC	1	7974			1.61 (0.87-2.94)[a]
	**Ever v Never (>40 years)**	Luminal A	1	7974			**1.39 (1.05-1.82)[a]**
		Luminal B	1	7974			1.25 (0.96-1.61)[a]
		HER2	1	7974			1.01 (0.61-1.67)[a]
		TNBC	1	7974			1.06 (0.65-1.75)[a]
	**≥24 v <12 months**	Luminal A	1	958			0.41 (0.13-1.39)[a]
		Luminal B	1	958			1.42 (0.51-4.62)[a]
		HER2	1	958			0.93 (0.27-4.26)[a]
		TNBC	1	958			0.76 (0.16-5.58)[a]
**Abortion**							
	**Ever v Never**	Luminal A	1	958		(24,107)	0.91 (0.34-2.17)[a]
		Luminal B	1	958			0.98 (0.56-1.66)[a]
		HER2	1	958			1.45 (0.71-2.88)[a]
		TNBC	1	958			0.85 (0.23-2.53)[a]
	**Induced v Never**	Luminal A	2	3962	0.618		**1.27 (1.04-1.50)**
		Luminal B	2	3962	0.520		1.03 (0.76-1.30)
		HER2	2	3962	0.654		1.23 (0.83-1.63)
		TNBC	2	3962	0.331		1.06 (0.80-1.32)
	**Spontaneous v Never**	Luminal A	2	3962	0.079		**0.60 (0.31-0.90)**
		Luminal B	2	3962	**0.018**		0.59 (0.09-1.28)
		HER2	2	3962	**0.014**		0.86 (0.34-2.06)
		TNBC	2	3962	0.164		0.82 (0.31-1.33)
**BMI (Standard)**							
	**Overweight/obese (≥25 v <18.5kg/m2)**	Luminal	1	2672		(9,34,120)	**2.96 (1.34-6.54)[a]**
		HER2	1	2672			**2.58 (1.60-4.14)[a]**
		TNBC	1	2672			1.27 (0.25-6.36)[a]
	**Overweight (25-29.9 v <18.5 kg/m2)**	ER+PR+	2	7389	0.286		1.03 (0.80-1.25)
		ER+PR-	2	7389	0.614		**0.81 (0.63-0.99)**
		ER-PR-	2	7389	0.463		0.84 (0.63-1.05)
	**Obese (≥30 v <18.5 kg/m2)**	ER+PR+	2	7389	**0.039**		1.48 (0.01-2.94)
		ER+PR-	2	7389	0.494		**0.56 (0.37-0.74)**
		ER-PR-	2	7389	0.167		**0.53 (0.33-0.73)**
**Alcohol**							
	**Past v Never**	ER+	1	50757		(53)	1.18 (0.72-1.93)[a]
		ER-	1	50757			**2.08 (1.12-3.87)[a]**
	**>150g/week v Never**	ER+	1	50757			1.58 (0.72-3.48)[a]
	**1-50g/week v Never**	ER-	1	50757			1.03 (0.50-2.13)[a]

Abbreviations: No, Number of studies; Total, sample size; I2(P), Heterogeneity test p-value; OR, Odds Ratio; RR, Risk Ratio/Relative Risk; 95% CI, 95% Confidence Interval; BMI, Body mass index; BMI Standard, European BMI classification.
[a] Effect sizes as reported in the respective studies.
Note: Findings tabulated based on five retrospective studies and a prospective study (alcohol intake).

Table S5: Breast cancer risk factors by location in Asian populations

Variable	Subgroup	No	Total	Reference	OR (95% CI)
**Parity**					
**1 v ≥4**	Urban	1	3152	(119)	1.39 (0.98-1.92)[a]
**1 v ≥4**	Rural	1	3152		**2.38 (1.33-4.17)[a]**
**Age at first birth**					
**≥26 v ≤20 years**	Urban	1	3152	(119)	**1.78 (1.32-2.41)[a]**
**≥26 v ≤20 years**	Rural	1	3152		**2.24 (1.13-4.43)[a]**
**Breastfeeding**					
**Never v Ever**	Urban	1	3152	(119)	0.98 (0.51-1.96)[a]
**Never v Ever**	Rural	1	3152		1.02 (0.25-4.00)[a]
**Abortion**					
**Induced v Never**	Urban	1	3152	(119)	**1.58 (1.15-2.16)[a]**
**Spontaneous v Never**		1	3152		**0.47 (0.29-0.75)[a]**
**Induced v Never**	Rural	1	3152		**2.08 (1.16-3.72)[a]**
**Spontaneous v Never**		1	3152		**0.42 (0.22-0.80)[a]**
**OC**					
**Ever v Never**	Urban	1	3152	(119)	1.28 (0.94-1.76)[a]
**Ever v Never**	Rural	1	3152		0.89 (0.52-1.52)[a]
**Sleep**					
**Less v More**	Urban	1	25	(119)	1.33 (0.95-1.89)[a]
**Less v More**	Rural	1	252		1.89 (0.88-4.17)[a]

Abbreviations: No, Number of studies; Total, sample size; OR, Odds Ratio; RR, Risk Ratio/Relative Risk; 95% CI, 95% Confidence Interval; OC, Oral contraceptives.
[a] Effect sizes as reported in the respective studies.
Note: Findings tabulated based on a retrospective study.

Table S6: Effect sizes, distributions, and attributable proportions of breast cancer risk factors in prospective studies among pre-menopausal Asian and European populations

Variable	OR/RR (95% CI)	Asian								OR/RR (95% CI)	European		P-value
		Singapore		South Korea		Taiwan		Japan					
		%	AR[b] (95% CI)	%	AR[b] (95% CI)	%	AR[b] (95% CI)	%	AR[b] (95% CI)		%	AR[c] (95% CI)	
**Pre-menopause**													
**Personal and family history**													
Family history of breast cancer (Yes v No)	**1.39 (1.22-1.59)**a					3.5	**1 (1-1)**			1.00 (0.68-1.47)	16.0	0 (0-0)	0.172
**Reproductive history**													
Parity (Nulliparous v Parous)	1.69 (0.72-2.66)					4.6	3 (3-3)	5.2	3 (3-3)	**1.41 (1.37-1.45)[a]**	20.1	**8 (8-8)**	0.956
Age at first birth													
23-25 v ≤25 years	**1.28 (1.18-1.39)[a]**					30.4	**8 (8-8)**						
26-30 v ≤25 years	1.28 (0.89-1.66)					29.7	8 (8-8)	29.1	8 (7-8)				
≥30 v ≤25 years	**1.70 (1.54-1.87)**					13.5	**9 (9-9)**	5.4	**4 (4-4)**	**1.24 (1.19-1.30)[a]**	37.5	**8 (8-8)**	0.587
Breastfeeding (Never v Ever)	**1.06 (1.01-1.11)**					44.7	**3 (3-3)**	11.3	**1 (1-1)**	0.88 (0.67-1.15)	25.0		0.104
Menarche													
<13 v ≥14 years	1.04 (0.63-1.75)[a]							16.1	1 (1-1)	0.99 (0.88-1.12)	44.0		0.818
<14 v ≥14 years	**1.22 (1.12-1.39)[a]**					28.3	**6 (6-6)**						
**Lifestyle and exogenous hormonal use**													
BMI (Asian, ≥24 v ≤23.9 kg/m^2)													
Overweight	0.98 (0.91-1.04)					24.0							
Obese	0.83 (0.49-1.17)					13.4							
Physical activity (Inactive v Active)	1.54 (0.80-2.28)							40.6	18 (18-18)	**1.30 (1.16-1.45)**	57.8	**15 (15-15)**	0.524
Sleep (≤6 v ≥8 hours)	1.11 (0.72-1.71)[a]							31.0	3 (3-3)				
OC (Ever v Never)	1.22 (0.96-1.55)[a]							9.7	2 (2-2)				
**Diet**													
Alcohol (Current v Never)	2.30 (0.97-5.45)[a]							4.1	5 (5-5)				
Meat (More v Less)	1.36 (0.77-2.43)[a]												
Dairy (More v Less)	1.20 (0.59-1.82)									**0.79 (0.63-0.99)**			0.475
Fish (Less vs More)	1.05 (0.59-1.89)[a]												
Fruits (Less v More)	0.81 (0.45-1.45)[a]									1.09 (0.83-1.41)			0.780
Vegetables (Less v More)	0.75 (0.42-1.33)[a]									**1.32 (1.05-1.67)**			0.594

Abbreviations: OR, Odds Ratio; RR, Risk Ratio/Relative Risk; 95% CI, Confidence Interval; Population, Population distribution; AR, Attributable risk; BMI, Body mass index; BMI Asian, Asian BMI classification; OC, Oral contraceptives.

[a] *Effect sizes as reported in the respective studies.*

[b] *AR values for Asians were determined using pooled effect sizes and risk factor distributions from each Asian country, with 95% confidence intervals determined using the delta method.*

[c] *AR values for Europeans were determined using effect sizes and risk factor distributions extracted from cohort studies, systematic reviews, meta-analyses, or collaborative projects, with 95% confidence intervals determined using the delta method.*

Note: P<0.05 are presented in bold. Sociodemographic factors are not included due to limited published effect sizes in the West for comparison. ΔP-value presented in this table are referring to effect size comparisons between populations. The AR values in the table indicate that when stated as X% of a risk factor, it represents the percentage of breast cancer cases attributed to that specific risk factor.

Table S7: Effect sizes, distributions, and attributable proportions of breast cancer risk factors in prospective studies among post-menopausal Asian and European populations

Variable	OR/RR (95% CI)	Asian								OR/RR (95% CI)	European		P-value
		Singapore		South Korea		Taiwan		Japan					
		%	AR[b] (95% CI)	%	AR[b] (95% CI)	%	AR[b] (95% CI)	%	AR[b] (95% CI)		%	AR[c] (95% CI)	
**Post-menopause**													
**Personal and family history**													
Family history of breast cancer (Yes v No)	**1.72 (1.51-1.92)**	2.4	**2 (2-2)**			3.2	**2 (2-2)**			0.96 (0.82-1.12)	16.7		**0.015**
**Reproductive history**													
Parity (Nulliparous v Parous)	**1.50 (1.20-1.81)**	7.2	**3 (3-3)**			4.0	**2 (2-2)**	6.6	**3 (3-3)**	**1.41 (1.37-1.45)[a]**	12.4	**5 (5-5)**	0.954
Age at first birth													
23-25 v ≤25 years	**1.15 (1.08-1.23)[a]**					33.3	**5 (5-5)**						
26-30 v ≤25 years	**1.34 (1.25-1.43)**					19.6	**6 (6-6)**	27.4	**9 (9-9)**				
≥30 v ≤25 years	**1.51 (1.35-1.67)**					7.3	**4 (4-4)**	5.1	**3 (3-3)**	**1.24 (1.19-1.30)**	17.8	**4 (4-4)**	0.299
Breastfeeding (Never v Ever)	**1.18 (1.10-1.125)**	30.0	**5 (5-5)**			22.4	**4 (4-4)**	8.6	**2 (2-2)**	1.12 (1.00-1.27)			
Menarche													
<13 v ≥14 years	1.22 (0.93-1.61)[a]							4.9	1 (1-1)				
<14 v ≥14 years	**1.28 (1.19-1.38)**	34.3	9 (9-9)			18.7	**5 (5-5)**						
Menopause													
46-48 v ≤45 years	0.97 (0.65-1.45)[a]			37.8									
49-51 v ≤45 years	1.36 (0.95-1.95)[a]			35.3	11 (11-11)								
50-54 v ≤44 years	1.23 (1.00-1.52)[a]							47.3	10 (10-10)				
>50 years v ≤45 years	**1.53 (1.11-1.96)**			6.0	**3 (3-3)**			5.2	**3 (3-3)**	**1.12 (1.07-1.17)**	54.0	**6 (6-6)**	**0.016**
**Lifestyle and exogenous hormonal use**													
BMI (Asian, ≥24 v ≤23.9 kg/m^2)													
Overweight	**1.19 (1.12-1.27)**					30.2	**5 (5-5)**						
Obese	**1.71 (1.31-2.11)**					19.7	**12 (12-12)**						
BMI (Standard, ≥25 v ≤24.9 kg/m^2)													
Overweight	**1.51 (1.21-1.88)[a]**	33.4	**15 (15-15)**							**1.12 (1.06-1.18)**	32.1	**4 (4-4)**	**0.008**
Obese	1.73 (0.83-3.59)[a]	10.1	7 (7-7)							**1.16 (1.08-1.25)**	21.7	**3 (3-3)**	**0.022**
Physical activity (Inactive v Active)	1.35 (0.85-1.85)							39.0	12 (12-12)	**1.15 (1.09-1.15)**	48.9	**7 (7-7)**	0.171
Smoking (Ever v Never)	0.80 (0.52-1.23)[a]	6.6											
Sleep (≤6 v ≥8 hours)	**1.98 (1.08-3.70)[a]**							29.1	**22 (22-22)**				
OC (Ever v Never)	1.02 (0.86-1.19)	37.3	1 (1-1)					8.3	0 (0-0)				
HRT													
Current v Never	**3.13 (1.37-7.12)[a]**					13.1	**22 (22-22)**			**1.08 (1.04-1.12)[a]**	17.0	**1 (1-1)**	**<0.001**
Former v Never	1.21 (0.45-3.27)[a]					1.2	0 (0-0)			**1.68 (1.64-1.72)[a]**	34.0	**19 (19-19)**	**0.015**
**Diet**													
Alcohol (Current v Never)	**2.74 (1.32-5.70)[a]**							2.6	**4 (4-4)**				
Meat (More v Less)	**3.06 (1.31-7.15)[a]**												
Dairy (More v Less)	1.38 (0.62-2.15)									0.92 (0.85-1.01)			0.080
Fish (Less vs More)	0.72 (0.29-1.82)[a]												
Fruits (Less v More)	0.82 (0.34-1.96)[a]									**1.12 (1.05-1.20)**			0.274
Vegetables (Less v More)	0.70 (0.30-1.61)[a]									0.97 (0.92-1.04)			0.212

Abbreviations: OR, Odds Ratio; RR, Risk Ratio/Relative Risk; 95% CI, Confidence Interval; Population, Population distribution; AR, Attributable risk; BMI, Body mass index; BMI Asian, Asian BMI classification; BMI Standard, European BMI classification; OC, Oral contraceptives; HRT, Hormone receptor therapy.

[a] Effect sizes as reported in the respective studies.

[b] AR values for Asians were determined using pooled effect sizes and risk factor distributions from each Asian country, with 95% confidence intervals determined using the delta method.

[c] AR values for Europeans were determined using effect sizes and risk factor distributions extracted from cohort studies, systematic reviews, meta-analyses, or collaborative projects, with 95% confidence intervals determined using the delta method.

Note: P<0.05 are presented in bold. Sociodemographic factors are not included due to limited published effect sizes in the West for comparison. ΔP-value presented in this table are referring to effect size comparisons between populations. The AR values in the table indicate that when stated as X% of a risk factor, it represents the percentage of breast cancer cases attributed to that specific risk factor.

Table S8: The source of estimates and distributions of breast cancer risk factors

Variable	Asian					European	
		Singapore	South Korea	Taiwan	Japan		
	Estimate	Distribution	Distribution	Distribution	Distribution	Estimate	Distribution
**Overall**							
**Personal and family history**							
Benign breast disease (Yes v No)	3.32 (39,110)	0.052 (110)	0.037 (42)			2.07 (129)	0.064 (130)
Family history of breast cancer (Yes v No)	2.30 (110)	0.025 (110)	0.034 (42)	0.033 (38)	0.014 (37)	3.90 (131)	0.164 (131)
**Reproductive history**							
Parity (Nulliparous v Parous)	1.28 (63,109,110)	0.072 (110)	0.066 (42)	0.042 (38)	0.088 (58)	1.15 (132)	0.158 (133)
Age at first birth (>30 v ≤30 years)	1.15 (110)	0.086 (110)					
Breastfeeding (Never v Ever)	1.17 (110)	0.304 (110)				1.08 (134)	0.370 (134)
Abortion (Yes v No)	1.01 (63)			0.031 (63)		0.97 (135)	0.268 (135)
Menarche							
<13 v ≥14 years	1.46 (40,109)		0.055 (40)		0.133 (58)	1.07 (136)	0.362 (136)
<14 v ≥14 years	1.26 (110)	0.349 (110)	0.197 (42)		0.278 (41)		
Menopause (Post- v Pre-menopause)	1.30 (109)		0.284 (42)				
**Lifestyle and exogenous hormonal use**							
BMI (Asian, ≥24 v ≤23.9 kg/m^2)							
Overweight	1.37 (65)				0.297 (56)		
Obese	1.86 (65)				0.032 (56)		
BMI (Standard, ≥25 v ≤24.9 kg/m^2)							
Overweight	1.45 (110)	0.333 (110)					
Obese	1.73 (110)	0.101 (110)					
Physical activity (Inactive v Active)	1.28 (55,61)				0.394 (48)	1.15 (137)	0.535 (138)
Smoking							
Current v Never	0.67 (58)				0.130 (58)	1.24 (139)	0.082 (139)
Former v Never	0.90 (58)				0.046 (58)	1.13 (32)	0.356 (139)
Passive smoking							
Current n Never	1.98 (58)				0.315 (58)	1.02 (140)	0.110 (140)
Former v Never	1.24 (58)				0.500 (58)	1.00 (89)	0.320 (140)
Sleep (≤6hours v ≥8 hours)	1.31 (37)				0.293 (37)		
OC (Ever v Never)	1.02 (109,110)	0.383 (110)	0.174 (42)		0.088 (62)	1.30 (132)	0.820 (132)
**Diet**							
Alcohol (Ever v Never)	0.50 (60,109)				0.279 (41)	1.46 (141)	0.580 (142)
Meat (More v Less)	1.20 (42,108)					1.08 (143)	
Dairy (More v Less)	1.32 (42)					0.89 (144)	
Fish (Less vs More)	0.98 (42,43)					0.99 (143)	
Fruits (Less v More)	0.79 (42,52)					1.09 (145)	
Vegetables (Less v More)	0.86 (42,52)					1.01 (145)	
Soy (Less v More)	1.01 (51,59)						
Isoflavone (Less v More)	1.01 (51,59)					1.00 (146)	
**Pre-menopause**							
**Personal and family history**							
Family history of breast cancer (Yes v No)	1.39 (38)			0.035 (38)		1.00 (131)	0.160 (133)
**Reproductive history**							
Parity (Nulliparous v Parous)	1.69 (3,38,62)			0.046 (38)	0.052 (62)	1.41 (147)	0.201 (133)
Age at first birth							
23-25 v ≤25 years	1.28 (38)			0.304 (38)			
26-30 v ≤25 years	1.28 (38,62)			0.297 (38)	0.291 (62)		
≥30 v ≤25 years	1.70 (38,62)			0.135 (38)	0.054 (62)	1.24 (147)	0.375 (133)
Breastfeeding (Never v Ever)	1.06 (38,62)			0.447 (38)	0.113 (62)	0.88 (134)	0.250 (132)
Menarche							

Variable	Asian					European	
	Estimate	Singapore Distribution	South Korea Distribution	Taiwan Distribution	Japan Distribution	Estimate	Distribution
**Overall**							
<13 v ≥14 years	1.04 (62)				0.161 (62)	0.99 (132)	0.440 (132)
<14 v ≥14 years	1.22 (38)			0.283 (38)			
**Lifestyle and exogenous hormonal use**							
BMI (Asian, ≥24 v ≤23.9 kg/m^2)							
Overweight	0.98 (38,56,65)			0.240 (38)			
Obese	0.83 (38,56,65)			0.134 (38)			
Physical activity (Inactive v Active)	1.54 (48,55)				0.406 (48)	1.30 (137)	0.578 (138)
Sleep (≤6 v ≥8 hours)	1.11 (37)				0.310 (37)		
OC (Ever v Never)	1.22 (62)				0.097 (62)		
**Diet**							
Alcohol (Current v Never)	2.30 (48)				0.041 (48)		
Meat (More v Less)	1.36 (42)						
Dairy (More v Less)	1.20 (42,44)					0.79 (144)	
Fish (Less vs More)	1.05 (42)						
Fruits (Less v More)	0.81 (42)					1.09 (145)	
Vegetables (Less v More)	0.75 (42)					1.32 (145)	
**Post-menopause**							
**Personal and family history**							
Family history of breast cancer (Yes v No)	1.72 (38,110)	0.024 (110)		0.032 (38)		0.96 (131)	0.167 (133)
**Reproductive history**							
Parity (Nulliparous v Parous)	1.50 (3,38,62)	0.072 (110)		0.040 (38)	0.066 (62)	1.41 (147)	0.124 (133)
Age at first birth							
23-25 v ≤25 years	1.15 (38)			0.333 (38)			
26-30 v ≤25 years	1.34 (38,62)			0.196 (38)	0.274 (62)		
≥30 v ≤25 years	1.51 (38,62)			0.073 (38)	0.051 (62)	1.24 (147)	0.178 (133)
Breastfeeding (Never v Ever)	1.18 (38,62,110)	0.300 (110)		0.224 (38)	0.086 (62)	1.12 (134)	
Menarche							
<13 v ≥14 years	1.22 (62)				0.049 (62)		
<14 v ≥14 years	1.28 (38,110)	0.343 (110)		0.187 (38)			
Menopause							
46-48 v ≤45 years	0.97 (9)		0.378 (9)				
49-51 v ≤45 years	1.36 (9)		0.353 (9)				
50-54 v ≤44 years	1.23 (62)				0.473 (62)		
>50 years v ≤45 years	1.53 (9,62)		0.060 (9)		0.052 (62)	1.12 (136)	0.540 (136)
**Lifestyle and exogenous hormonal use**							
BMI (Asian, ≥24 v ≤23.9 kg/m^2)							
Overweight	1.19 (38,56,65)			0.302 (38)			
Obese	1.71 (38,56,65)			0.197 (38)			
BMI (Standard, ≥25 v ≤24.9 kg/m^2)							
Overweight	1.51 (110)	0.334 (110)				1.12 (148)	0.321 (148)
Obese	1.73 (110)	0.101 (110)				1.16 (148)	0.217 (148)
Physical activity (Inactive v Active)	1.35 (48,55)				0.390 (48)	1.15 (137)	0.489 (138)
Smoking (Ever v Never)	0.80 (110)	0.066 (110)					
Sleep (≤6 v ≥8 hours)	1.98 (37)				0.291 (37)		
OC (Ever v Never)	1.02 (62,110)	0.373 (110)			0.083 (62)		
HRT							
Current v Never	3.13 (45)			0.131 (45)		1.08 (149)	0.170 (133)
Former v Never	1.21 (45)			0.012 (45)		1.68 (149)	0.340 (133)
**Diet**							
Alcohol (Current v Never)	2.74 (48)				0.026 (48)		

Variable	Asian					European	
		Singapore	South Korea	Taiwan	Japan		
	Estimate	Distribution	Distribution	Distribution	Distribution	Estimate	Distribution
**Overall**							
Meat (More v Less)	3.06 (42)						
Dairy (More v Less)	1.38 (42,44)					0.92 (144)	
Fish (Less vs More)	0.72 (42)						
Fruits (Less v More)	0.82 (42)					1.12 (145)	
Vegetables (Less v More)	0.70 (42)					0.97 (145)	

Abbreviations: BMI, Body mass index; BMI Asian, Asian BMI classification; BMI Standard, European BMI classification; OC, Oral contraceptives; HRT, Hormone receptor therapy.
Note: Citations are indicated within parentheses immediately following the respective data points.

SUPPLEMENTAL REFERENCES

1. Chang Y-J, Hou Y-C, Chen L-J, Wu J-H, Wu C-C, Chang Y-J*, et al.* Is vegetarian diet associated with a lower risk of breast cancer in Taiwanese women? BMC public health **2017**;17:1-9.
2. Cho Y, Kim J, Park K, Lim S, Shin A, Sung M*, et al.* Effect of dietary soy intake on breast cancer risk according to menopause and hormone receptor status. European journal of clinical nutrition **2010**;64(9):924-32.
3. Fu X, Shi X, Lin K, Lin H, Huang W, Zhang G*, et al.* Environmental and DNA repair risk factors for breast cancer in South China. International Journal of Hygiene and Environmental Health **2015**;218(3):313-8.
4. Gao C-M, Ding J-H, Li S-P, Liu Y-T, Qian Y, Chang J*, et al.* Active and passive smoking, and alcohol drinking and breast cancer risk in Chinese women. Asian Pacific Journal of Cancer Prevention **2013**;14(2):993-6.
5. Islam T, Ito H, Sueta A, Hosono S, Hirose K, Watanabe M*, et al.* Alcohol and dietary folate intake and the risk of breast cancer. European journal of cancer prevention **2013**;22(4):358-66.
6. Islam T, Matsuo K, Ito H, Hosono S, Watanabe M, Iwata H*, et al.* Reproductive and hormonal risk factors for luminal, HER2-overexpressing, and triple-negative breast cancer in Japanese women. Annals of oncology **2012**;23(9):2435-41.
7. Itoh H, Iwasaki M, Sawada N, Takachi R, Kasuga Y, Yokoyama S*, et al.* Dietary cadmium intake and breast cancer risk in Japanese women: a case–control study. International journal of hygiene and environmental health **2014**;217(1):70-7.
8. Iwasaki M, Mizusawa J, Kasuga Y, Yokoyama S, Onuma H, Nishimura H*, et al.* Green tea consumption and breast cancer risk in Japanese women: a case-control study. Nutrition and cancer **2014**;66(1):57-67.
9. Kawai M, Kakugawa Y, Nishino Y, Hamanaka Y, Ohuchi N, Minami Y. Anthropometric factors, physical activity, and breast cancer risk in relation to hormone receptor and menopausal status in Japanese women: a case–control study. Cancer Causes & Control **2013**;24:1033-44.
10. Kawase T, Matsuo K, Suzuki T, Hirose K, Hosono S, Watanabe M*, et al.* Association between vitamin D and calcium intake and breast cancer risk according to menopausal status and receptor status in Japan. Cancer science **2010**;101(5):1234-40.
11. Li A, Shen Z, Sun Z, Yun S, Tian X, Hu Z*, et al.* Occupational risk factors and breast cancer in Beijing, China: a hospital-based case–control study. BMJ open **2022**;12(2):e054151.
12. Lu S, Qian Y, Huang X, Yu H, Yang J, Han R*, et al.* The association of dietary pattern and breast cancer in Jiangsu, China: A population-based case-control study. PLoS One **2017**;12(9):e0184453.
13. Mizoo T, Taira N, Nishiyama K, Nogami T, Iwamoto T, Motoki T*, et al.* Effects of lifestyle and single nucleotide polymorphisms on breast cancer risk: a case–control study in Japanese women. BMC cancer **2013**;13:1-15.
14. Nagata C, Nagao Y, Nakamura K, Wada K, Tamai Y, Tsuji M*, et al.* Cadmium exposure and the risk of breast cancer in Japanese women. Breast cancer research and treatment **2013**;138:235-9.
15. Takizawa Y, Kawai M, Kakugawa Y, Nishino Y, Ohuchi N, Minami Y. Alcohol consumption and breast cancer risk according to hormone receptor status in Japanese women: A case-control study. The Tohoku Journal of Experimental Medicine **2018**;244(1):63-73.
16. Toi M, Hirota S, Tomotaki A, Sato N, Hozumi Y, Anan K*, et al.* Probiotic beverage with soy isoflavone consumption for breast cancer prevention: a case-control study. Current Nutrition & Food Science **2013**;9(3):194-200.
17. Tong J-h, Li Z, Shi J, Li H-m, Wang Y, Fu L-y*, et al.* Passive smoking exposure from partners as a risk factor for ER+/PR+ double positive breast cancer in never-smoking Chinese urban women: a hospital-based matched case control study. Plos one **2014**;9(5):e97498.
18. Tse LA, Li M, Chan W-c, Kwok C-h, Leung S-l, Wu C*, et al.* Familial risks and estrogen receptor-positive breast cancer in Hong Kong Chinese women. PLoS One **2015**;10(3):e0120741.
19. Wang F, Dai J, Li M, Chan W-c, Kwok CC-h, Leung S-l*, et al.* Risk assessment model for invasive breast cancer in Hong Kong women. Medicine **2016**;95(32).
20. Wang JM, Wang J, Zhao HG, Liu TT, Wang FY. Reproductive risk factors associated with breast cancer molecular subtypes among young women in Northern China. BioMed Research International **2020**;2020.

21. Wang L, Liu L, Lou Z, Ding L, Guan H, Wang F*, et al.* Risk prediction for breast Cancer in Han Chinese women based on a cause-specific Hazard model. BMC cancer **2019**;19(1):1-8.
22. Wang X-L, Jia C-X, Liu L-Y, Zhang Q, Li Y-Y, Li L. Obesity, diabetes mellitus, and the risk of female breast cancer in Eastern China. World Journal of Surgical Oncology **2013**;11:1-7.
23. Wu J-Q, Li Y-Y, Ren J-C, Zhao R, Zhou Y, Gao E-S. Induced abortion and breast cancer: results from a population-based case control study in China. Asian Pacific Journal of Cancer Prevention **2014**;15(8):3635-40.
24. Xing P, Li J, Jin F. A case–control study of reproductive factors associated with subtypes of breast cancer in Northeast China. Medical oncology **2010**;27:926-31.
25. Xu J, Qiu X, Li Y, Sun N, Zhang Y, Shu J. Hyperlipoproteinemia (a) is associated with breast cancer in a Han Chinese population. Medicine **2020**;99(38).
26. Yu H, Hwang J-Y, Ro J, Kim J, Chang N. Vegetables, but not pickled vegetables, are negatively associated with the risk of breast cancer. Nutrition and cancer **2010**;62(4):443-53.
27. Yuan X, Yi F, Hou C, Lee H, Zhong X, Tao P*, et al.* Induced abortion, birth control methods, and breast cancer risk: A case-control study in China. Journal of Epidemiology **2019**;29(5):173-9.
28. Zhang C, Ho SC, Lin F, Cheng S, Fu J, Chen Y. Soy product and isoflavone intake and breast cancer risk defined by hormone receptor status. Cancer science **2010**;101(2):501-7.
29. Zhang M, Holman C. Low-to-moderate alcohol intake and breast cancer risk in Chinese women. British journal of cancer **2011**;105(7):1089-95.
30. Zhu Y-Y, Zhou L, Jiao S-c, Xu L-z. Relationship between soy food intake and breast cancer in China. Asian Pac J Cancer Prev **2011**;12(11):2837-40.
31. Liu Y-T, Gao C-M, Ding J-H, Li S-P, Cao H-X, Wu J-Z*, et al.* Physiological, reproductive factors and breast cancer risk in Jiangsu province of China. Asian Pac J Cancer Prev **2011**;12(3):787-90.
32. Huang M-C, Huang T-T, Feng H-C, Chen I-C, Chang C-I, Wang T-N*, et al.* Lifestyle Factors and Energy Intakes with Risks of Breast Cancer among Pre-and Post-Menopausal Women in Taiwan. Nutrients **2023**;15(18):3900.
33. Park S, Lee DH, Jeon JY, Ryu J, Kim S, Kim JY*, et al.* Serum 25-hydroxyvitamin D deficiency and increased risk of breast cancer among Korean women: a case–control study. Breast Cancer Research and Treatment **2015**;152:147-54.
34. Li H, Sun X, Miller E, Wang Q, Tao P, Liu L*, et al.* BMI, reproductive factors, and breast cancer molecular subtypes: A case-control study and meta-analysis. Journal of epidemiology **2017**;27(4):143-51.
35. Liu L-Y, Wang F, Cui S-D, Tian F-G, Fan Z-M, Geng C-Z*, et al.* A case-control study on risk factors of breast cancer in Han Chinese women. Oncotarget **2017**;8(57):97217.
36. Lee M-S, Huang Y-C, Wahlqvist ML, Wu T-Y, Chou Y-C, Wu M-H*, et al.* Vitamin D decreases risk of breast cancer in premenopausal women of normal weight in subtropical Taiwan. Journal of Epidemiology **2011**;21(2):87-94.
37. Cao J, Eshak ES, Liu K, Muraki I, Cui R, Iso H*, et al.* Sleep duration and risk of breast cancer: The JACC Study. Breast cancer research and treatment **2019**;174:219-25.
38. Chen M, Wu WY, Yen AM, Fann JC, Chen SL, Chiu SY*, et al.* Body mass index and breast cancer: analysis of a nation-wide population-based prospective cohort study on 1 393 985 Taiwanese women. International Journal of Obesity **2016**;40(3):524-30.
39. Chuang S-C, Wu G-J, Lu Y-S, Lin C-H, Hsiung CA. Associations between medical conditions and breast cancer risk in Asians: a nationwide population-based study in Taiwan. PloS one **2015**;10(11):e0143410.
40. Jung KJ, Park C, Yun YD, Jee SH. Duration of ovarian hormone exposure and gynecological cancer risk in Korean women: the Korean Heart Study. Cancer epidemiology **2016**;41:1-7.
41. Kawai M, Minami Y, Kakizaki M, Kakugawa Y, Nishino Y, Fukao A*, et al.* Alcohol consumption and breast cancer risk in Japanese women: the Miyagi Cohort study. Breast cancer research and treatment **2011**;128:817-25.
42. Kim JH, Lee J, Jung S-Y, Kim J. Dietary factors and female breast cancer risk: a prospective cohort study. Nutrients **2017**;9(12):1331.
43. Kiyabu GY, Inoue M, Saito E, Abe SK, Sawada N, Ishihara J*, et al.* Fish, n− 3 polyunsaturated fatty acids and n− 6 polyunsaturated fatty acids intake and breast cancer risk: The J apan P ublic H ealth C enter-based prospective study. International journal of cancer **2015**;137(12):2915-26.

44. Kojima R, Okada E, Ukawa S, Mori M, Wakai K, Date C*, et al.* Dietary patterns and breast cancer risk in a prospective Japanese study. Breast cancer **2017**;24(1):152-60.
45. Lai J-N, Wu C-T, Chen P-C, Huang C-S, Chow S-N, Wang J-D. Increased risk for invasive breast cancer associated with hormonal therapy: a nation-wide random sample of 65,723 women followed from 1997 to 2008. PloS one **2011**;6(10):e25183.
46. Li W, Ray RM, Thomas DB, Davis S, Yost M, Breslow N*, et al.* Shift work and breast cancer among women textile workers in Shanghai, China. Cancer Causes & Control **2015**;26:143-50.
47. Liu Y, Warren Andersen S, Wen W, Gao YT, Lan Q, Rothman N*, et al.* Prospective cohort study of general and central obesity, weight change trajectory and risk of major cancers among Chinese women. International journal of cancer **2016**;139(7):1461-70.
48. Nitta J, Nojima M, Ohnishi H, Mori M, Wakai K, Suzuki S*, et al.* Weight gain and alcohol drinking associations with breast cancer risk in Japanese postmenopausal women-results from the Japan Collaborative Cohort (JACC) Study. Asian Pacific Journal of Cancer Prevention **2016**;17(3):1437-43.
49. Sari GN, Eshak ES, Shirai K, Fujino Y, Tamakoshi A, Iso H. Association of job category and occupational activity with breast cancer incidence in Japanese female workers: the JACC study. BMC Public Health **2020**;20(1):1-10.
50. Shin S, Saito E, Inoue M, Sawada N, Ishihara J, Takachi R*, et al.* Dietary pattern and breast cancer risk in Japanese women: the Japan Public Health Center-based Prospective Study (JPHC Study). British journal of nutrition **2016**;115(10):1769-79.
51. Shirabe R, Saito E, Sawada N, Ishihara J, Takachi R, Abe SK*, et al.* Fermented and nonfermented soy foods and the risk of breast cancer in a Japanese population-based cohort study. Cancer Medicine **2021**;10(2):757-71.
52. Suzuki R, Iwasaki M, Hara A, Inoue M, Sasazuki S, Sawada N*, et al.* Fruit and vegetable intake and breast cancer risk defined by estrogen and progesterone receptor status: the Japan Public Health Center-based Prospective Study. Cancer Causes & Control **2013**;24:2117-28.
53. Suzuki R, Iwasaki M, Inoue M, Sasazuki S, Sawada N, Yamaji T*, et al.* Alcohol consumption-associated breast cancer incidence and potential effect modifiers: the Japan Public Health Center-based Prospective Study. International journal of cancer **2010**;127(3):685-95.
54. Suzuki R, Iwasaki M, Inoue M, Sasazuki S, Sawada N, Yamaji T*, et al.* Body weight at age 20 years, subsequent weight change and breast cancer risk defined by estrogen and progesterone receptor status—the Japan public health center-based prospective study. International Journal of Cancer **2011**;129(5):1214-24.
55. Suzuki R, Iwasaki M, Yamamoto S, Inoue M, Sasazuki S, Sawada N*, et al.* Leisure-time physical activity and breast cancer risk defined by estrogen and progesterone receptor status—the Japan Public Health Center-based Prospective Study. Preventive medicine **2011**;52(3-4):227-33.
56. Suzuki S, Kojima M, Tokudome S, Mori M, Sakauchi F, Wakai K*, et al.* Obesity/weight gain and breast cancer risk: findings from the Japan collaborative cohort study for the evaluation of cancer risk. Journal of epidemiology **2013**;23(2):139-45.
57. Suzuki Y, Tsunoda H, Kimura T, Yamauchi H. BMI change and abdominal circumference are risk factors for breast cancer, even in Asian women. Breast Cancer research and treatment **2017**;166:919-25.
58. Wada K, Kawachi T, Hori A, Takeyama N, Tanabashi S, Matsushita S*, et al.* Husband's smoking status and breast cancer risk in Japan: From the Takayama study. Cancer Science **2015**;106(4):455-60.
59. Wada K, Nakamura K, Tamai Y, Tsuji M, Kawachi T, Hori A*, et al.* Soy isoflavone intake and breast cancer risk in Japan: from the Takayama study. International Journal of Cancer **2013**;133(4):952-60.
60. Wang Y-C, Lin C-H, Huang S-P, Chen M, Lee T-S. Risk factors for female breast cancer: a population cohort study. Cancers **2022**;14(3):788.
61. Su J, Jiang Y, Fan X, Tao R, Wu M, Lu Y*, et al.* Association between physical activity and cancer risk among Chinese adults: a 10-year prospective study. International Journal of Behavioral Nutrition and Physical Activity **2022**;19(1):1-8.
62. Takeuchi T, Kitamura Y, Sobue T, Utada M, Ozasa K, Sugawara Y*, et al.* Impact of reproductive factors on breast cancer incidence: Pooled analysis of nine cohort studies in Japan. Cancer Medicine **2021**;10(6):2153-63.

63. Shen C-T, Tai S-Y, Tsao Y-H, Chen F-M, Hsieh H-M. Abortion and Female Cancer Risks among Women Aged 20 to 45 Years: A 10-Year Longitudinal Population-Based Cohort Study in Taiwan. International Journal of Environmental Research and Public Health **2023**;20(4):3682.
64. Park B, Kim S, Kim H, Cha C, Chung MS. Associations between obesity, metabolic health, and the risk of breast cancer in East Asian women. British Journal of Cancer **2021**;125(12):1718-25.
65. Guo L, Li N, Wang G, Su K, Li F, Yang L*, et al.* Body mass index and cancer incidence: a prospective cohort study in northern China. Zhonghua liu xing bing xue za zhi= Zhonghua liuxingbingxue zazhi **2014**;35(3):231-6.
66. Akbari A, Khayamzadeh M, Akbari ME, Sohrabi MR, Ajori L. The Relationship of Pre and Early Postnatal Risk Factors with Breast Cancer. Asian Pacific Journal of Cancer Prevention: APJCP **2020**;21(1):75.
67. Dianatinasab M, Fararouei M, Mohammadianpanah M, Zare-Bandamiri M, Rezaianzadeh A. Hair coloring, stress, and smoking increase the risk of breast cancer: a case-control study. Clinical breast cancer **2017**;17(8):650-9.
68. Elkum N, Al-Tweigeri T, Ajarim D, Al-Zahrani A, Amer SMB, Aboussekhra A. Obesity is a significant risk factor for breast cancer in Arab women. BMC cancer **2014**;14(1):1-10.
69. Ghiasvand R, Bahmanyar S, Zendehdel K, Tahmasebi S, Talei A, Adami H-O*, et al.* Postmenopausal breast cancer in Iran; risk factors and their population attributable fractions. BMC cancer **2012**;12(1):1-9.
70. Ghiasvand R, Maram ES, Tahmasebi S, Tabatabaee SHR. Risk factors for breast cancer among young women in southern Iran. International journal of cancer **2011**;129(6):1443-9.
71. Hajian-Tilaki K, Gholizadehpasha A, Bozorgzadeh S, Hajian-Tilaki E. Body mass index and waist circumference are predictor biomarkers of breast cancer risk in Iranian women. Medical oncology **2011**;28:1296-301.
72. Hajian-Tilaki K, Kaveh-Ahangar T. Reproductive factors associated with breast cancer risk in northern Iran. Medical Oncology **2011**;28:441-6.
73. Hajian-Tilaki K, Kaveh-Ahangar T, Hajian-Tilaki E. Is educational level associated with breast cancer risk in Iranian women? Breast cancer **2012**;19:64-70.
74. Heidari Z, Jalali S, Sedaghat F, Ehteshami M, Rashidkhani B. Dietary patterns and breast cancer risk among Iranian women: a case-control study. European Journal of Obstetrics & Gynecology and Reproductive Biology **2018**;230:73-8.
75. Jamshidinaeini Y, Akbari ME, Abdollahi M, Ajami M, Davoodi SH. Vitamin D status and risk of breast cancer in Iranian women: a case–control study. Journal of the American College of Nutrition **2016**;35(7):639-46.
76. Joukar F, Ahmadnia Z, Atrkar-Roushan Z, Hasavari F, Rahimi A. The investigation of risk factors impacting breast cancer in Guilan Province. Asian Pacific journal of cancer prevention: APJCP **2016**;17(10):4623.
77. Keshet-Sitton A, Or-Chen K, Yitzhak S, Tzabary I, Haim A. Light and the city: breast cancer risk factors differ between urban and rural women in Israel. Integrative cancer therapies **2017**;16(2):176-87.
78. Khachatryan L, Scharpf R, Kagan S. Influence of diabetes mellitus type 2 and prolonged estrogen exposure on risk of breast cancer among women in Armenia. Health care for women international **2011**;32(11):953-71.
79. Maleki F, Fotouhi A, Ghiasvand R, Harirchi I, Talebi G, Rostami S*, et al.* Association of physical activity, body mass index and reproductive history with breast cancer by menopausal status in Iranian women. Cancer Epidemiology **2020**;67:101738.
80. Marzbani B, Nazari J, Najafi F, Marzbani B, Shahabadi S, Amini M*, et al.* Dietary patterns, nutrition, and risk of breast cancer: a case-control study in the west of Iran. Epidemiology and health **2019**;41.
81. Safabakhsh M, Imani H, Shab-Bidar S. Higher dietary total antioxidant capacity is not associated with risk of breast cancer in Iranian women. Breast Cancer **2020**;27:652-61.
82. Safabakhsh M, Shab-Bidar S, Imani H. Higher Fruits and Vegetables Consumption Is not Associated with Risk of Breast Cancer in Iranian Women. Nutrition and Cancer **2022**;74(5):1680-91.
83. Sepandi M, Akrami M, Tabatabaee H, Rajaeefard A, Tahmasebi S, Angali KA*, et al.* Breast cancer risk factors in women participating in a breast screening program: a study on 11,850 Iranian females. Asian Pacific Journal of Cancer Prevention **2014**;15(19):8499-502.
84. Sezer H, Yilmaz M, Gurler H, Koyuncu A. Breast cancer risk factors in Turkey: a hospital-based case-control study. Asian Pac J Cancer Prev **2011**;12(9):2317-22.

85. Naz S, Masroor I, Akhtar W, Abrar S, Saeed SA, Sajjad Z. Evaluation of common risk factors for breast carcinoma in females: a hospital based study in Karachi, Pakistan. Asian Pacific Journal of Cancer Prevention **2015**;16(1):6347.
86. Tajaddini A, Pourzand A, Sanaat Z, Pirouzpanah S. Dietary resistant starch contained foods and breast cancer risk: a case-control study in northwest of Iran. Asian Pacific Journal of Cancer Prevention **2015**;16(10):4185-92.
87. Tehranian N, Shobeiri F, Pour FH, Hagizadeh E. Risk factors for breast cancer in Iranian women aged less than 40 years. Asian Pac J Cancer Prev **2010**;11(6):1723-5.
88. Yousef FM, Jacobs ET, Kang PT, Hakim IA, Going S, Yousef JM*, et al.* Vitamin D status and breast cancer in Saudi Arabian women: case-control study. The American journal of clinical nutrition **2013**;98(1):105-10.
89. Veisy A, Lotfinejad S, Salehi K, Zhian F. Risk of breast cancer in relation to reproductive factors in North-West of Iran, 2013-2014. Asian Pacific Journal of Cancer Prevention **2015**;16(2):451-5.
90. Bashamakha G, bin Sumait H, Bashamakha M, Al Serouri A, Khader Y. Risk factors of breast cancer in Hadramout Valley and Desert, Yemen. International Journal of Preventive Medicine **2019**;10.
91. Alsolami FJ, Azzeh FS, Ghafouri KJ, Ghaith MM, Almaimani RA, Almasmoum HA*, et al.* Determinants of breast cancer in Saudi women from Makkah region: a case-control study (breast cancer risk factors among Saudi women). BMC public health **2019**;19:1-8.
92. El Sharif N, Khatib I. Reproductive factors and breast cancer risk in Palestine: A case control study. Cancer Epidemiology **2021**;74:102019.
93. Al Qadire M, Alkhalaileh M, Hedaya H. Risk factors for breast Cancer among Jordanian women: a case-control study. Iranian Journal of Public Health **2018**;47(1):49.
94. Goldberg M, Calderon-Margalit R, Paltiel O, Abu Ahmad W, Friedlander Y, Harlap S*, et al.* Socioeconomic disparities in breast cancer incidence and survival among parous women: findings from a population-based cohort, 1964–2008. BMC cancer **2015**;15:1-11.
95. Chaveepojnkamjorn W, Thotong R, Sativipawee P, Pitikultang S. Body mass index and breast cancer risk among Thai premenopausal women: A case-control study. Asian Pacific Journal of Cancer Prevention: APJCP **2017**;18(11):3097.
96. Ekpanyaskul C, Khuhaprema T, Wiangnon S, Sangrajrang S. Case-control study of occupational categories and breast cancer risk in Thailand. Asian Pac J Cancer Prev **2010**;11(3):793-7.
97. Matalqah L, Radaideh K, Yusoff ZM, Awaisu A. Predictors of breast cancer among women in a northern state of Malaysia: a matched case-control study. Asian Pac J Cancer Prev **2011**;12(6):1549-53.
98. Nguyen J, Le Q, Duong B, Sun P, Pham H, Ta V*, et al.* A matched case-control study of risk factors for breast cancer risk in Vietnam. International journal of breast cancer **2016**;2016.
99. Razif SM, Sulaiman S, Hanie SS, Aina EN, Rohaizak M, Fuad I*, et al.* The contribution of reproductive factors and family history towards premenopausal breast cancer risk in Kuala Lumpur, Malaysia. Med J Malaysia **2011**;66(3):220-6.
100. Shahar S, Salleh RM, Ghazali AR, Koon PB, Mohamud W. Roles of adiposity, lifetime physical activity and serum adiponectin in occurrence of breast cancer among Malaysian women in Klang Valley. Asian Pac J Cancer Prev **2010**;11(1):61-6.
101. Shahril MR, Sulaiman S, Shaharudin SH, Akmal SN. Healthy eating index and breast cancer risk among Malaysian women. European Journal of Cancer Prevention **2013**;22(4):342-7.
102. Tan M-M, Ho W-K, Yoon S-Y, Mariapun S, Hasan SN, Lee DS-C*, et al.* A case-control study of breast cancer risk factors in 7,663 women in Malaysia. PloS one **2018**;13(9):e0203469.
103. Trieu PDY, Mello-Thoms C, Peat JK, Do TD, Brennan PC. Risk factors of female breast cancer in Vietnam: a case-control study. Cancer research and treatment: Official journal of Korean Cancer Association **2017**;49(4):990-1000.
104. Wahidin M, Djuwita R, Adisasmita A. Oral contraceptive and breast cancer risks: A case control study in six referral hospitals in Indonesia. Asian Pacific journal of cancer prevention: APJCP **2018**;19(8):2199.
105. Yen SH, Knight A, Krishna M, Muda W, Rufai A. Lifetime physical activity and breast cancer a case-control study in Kelantan, Malaysia. Asian Pacific Journal of Cancer Prevention **2016**;17(8):4083-8.

106. Sulaiman S, Shahril MR, Shaharudin SH, Emran NA, Muhammad R, Ismail F*, et al.* Fat intake and its relationship with pre-and postmenopausal breast cancer risk: a case-control study in Malaysia. Asian Pac J Cancer Prev **2011**;12(9):2167-78.
107. Bui OT, Tran HT, Nguyen SM, Dao TV, Bui QV, Pham AT*, et al.* Menstrual and reproductive factors in association with breast cancer risk in Vietnamese women: a case-control study. Cancer Control **2022**;29:10732748221140206.
108. Butler LM, Wu AH, Wang R, Koh W-P, Yuan J-M, Yu MC. A vegetable-fruit-soy dietary pattern protects against breast cancer among postmenopausal Singapore Chinese women. The American journal of clinical nutrition **2010**;91(4):1013-9.
109. Gibson LJ, Héry C, Mitton N, Gines-Bautista A, Parkin DM, Ngelangel C*, et al.* Risk factors for breast cancer among Filipino women in Manila. International journal of cancer **2010**;126(2):515-21.
110. Ho PJ, Lau HSH, Ho WK, Wong FY, Yang Q, Tan KW*, et al.* Incidence of breast cancer attributable to breast density, modifiable and non-modifiable breast cancer risk factors in Singapore. Scientific reports **2020**;10(1):503.
111. Bano R, Ismail M, Nadeem A, Khan MH, Rashid H. Potential risk factors for breast cancer in Pakistani women. Asian Pacific Journal of Cancer Prevention **2016**;17(9):4307-12.
112. Das S, Sen S, Mukherjee A, Chakraborty D, Mondal PK. Risk factors of breast cancer among women in eastern India: a tertiary hospital based case control study. Asian Pacific Journal of Cancer Prevention **2012**;13(10):4979-81.
113. Gathani T, Barnes I, Ali R, Arumugham R, Chacko R, Digumarti R*, et al.* Lifelong vegetarianism and breast cancer risk: a large multicentre case control study in India. BMC Women's Health **2017**;17(1):1-6.
114. Haseen SD, Khanam A, Sultan N, Idrees F, Akhtar N, Imtiaz F. Elevated fasting blood glucose is associated with increased risk of breast cancer: outcome of case-control study conducted in Karachi, Pakistan. Asian Pacific Journal of Cancer Prevention **2015**;16(2):675-8.
115. Bhadoria A, Kapil U, Sareen N, Singh P. Reproductive factors and breast cancer: A case—control study in tertiary care hospital of North India. Indian Journal of Cancer **2013**;50(4):316-21.
116. Lodha R, Joshi A, Paul D, Lodha K, Nahar N, Shrivastava A*, et al.* Association between reproductive factors and breast cancer in an urban set up at central India: A case-control study. Indian Journal of Cancer **2011**;48(3):303-7.
117. Lodha RS, Nandeshwar S, Pal D, Shrivastav A, Lodha K, Bhagat VK*, et al.* Risk factors for breast cancer among women in Bhopal urban agglomerate: a case-control study. Asian Pac J Cancer Prev **2011**;12(8):2111-5.
118. Mohite VR, Pratinidhi AK, Mohite RV. Reproductive risk factors and breast cancer: a case control study from rural India. Bangladesh Journal of Medical Science **2015**;14(3):258-64.
119. Nagrani R, Mhatre S, Boffetta P, Rajaraman P, Badwe R, Gupta S*, et al.* Understanding rural–urban differences in risk factors for breast cancer in an Indian population. Cancer Causes & Control **2016**;27:199-208.
120. Nagrani R, Mhatre S, Rajaraman P, Soerjomataram I, Boffetta P, Gupta S*, et al.* Central obesity increases risk of breast cancer irrespective of menopausal and hormonal receptor status in women of South Asian Ethnicity. European Journal of Cancer **2016**;66:153-61.
121. Rajbongshi N, Mahanta L, Nath D, Sarma J. A matched case control study of risk indicators of breast cancer in Assam, India. Mymensingh Med J **2015**;24(2):385-91.
122. Shamsi U, Khan S, Azam I, Habib Khan A, Maqbool A, Hanif M*, et al.* A multicenter case control study of association of vitamin D with breast cancer among women in Karachi, Pakistan. PLoS One **2020**;15(1):e0225402.
123. Shamsi U, Khan S, Usman S, Soomro S, Azam I. A multicenter matched case control study of breast cancer risk factors among women in Karachi, Pakistan. Asian Pacific Journal of Cancer Prevention **2013**;14(1):183-8.
124. Shridhar K, Singh G, Dey S, Singh Dhatt S, Paul Singh Gill J, Goodman M*, et al.* Dietary patterns and breast cancer risk: a multi-centre case control study among North Indian Women. International journal of environmental research and public health **2018**;15(9):1946.
125. Vishwakarma G, Mehta A, Saifi M, Garg D, Paliwal D. Modifiable (Sleeping Pattern and Stress) and Non-Modifiable Risk Factors Associated with Breast Cancer: A Matched Case-Control Study in Delhi, India. Asian Pacific Journal of Cancer Prevention **2022**;23(7):2469-76.

126. Shetty V, Kundapur R, Chandramohan S, Baisil S, Saxena D. Dietary risk with other risk factors of breast cancer. Indian Journal of Community Medicine: Official Publication of Indian Association of Preventive & Social Medicine **2021**;46(3):396.
127. Toleutay U, Reznik V, Kalmatayeva Z, Smigelskas K. Risk factors of breast cancer in Kyzylorda oblast of Kazakhstan: a case-control study. Asian Pacific Journal of Cancer Prevention **2013**;14(10):5961-4.
128. Baset Z, Abdul-Ghafar J, Parpio YN, Haidary AM. Risk factors of breast cancer among patients in a tertiary care hospitals in Afghanistan: a case control study. BMC cancer **2021**;21:1-9.
129. Dyrstad SW, Yan Y, Fowler AM, Colditz GA. Breast cancer risk associated with benign breast disease: systematic review and meta-analysis. Breast cancer research and treatment **2015**;149:569-75.
130. Ashbeck EL, Rosenberg RD, Stauber PM, Key CR. Benign breast biopsy diagnosis and subsequent risk of breast cancer. Cancer epidemiology, biomarkers & prevention **2007**;16(3):467-72.
131. Cancer CGoHFiB. Familial breast cancer: collaborative reanalysis of individual data from 52 epidemiological studies including 58 209 women with breast cancer and 101 986 women without the disease. The Lancet **2001**;358(9291):1389-99.
132. Nelson HD, Zakher B, Cantor A, Fu R, Griffin J, O'Meara ES*, et al.* Risk factors for breast cancer for women aged 40 to 49 years: a systematic review and meta-analysis. Annals of internal medicine **2012**;156(9):635-48.
133. Pal Choudhury P, Wilcox AN, Brook MN, Zhang Y, Ahearn T, Orr N*, et al.* Comparative validation of breast cancer risk prediction models and projections for future risk stratification. JNCI: Journal of the National Cancer Institute **2020**;112(3):278-85.
134. Michels KB, Willett WC, Hunter D, Colditz G, Rosner B, Manson J*, et al.* Prospective assessment of breastfeeding and breast cancer incidence among 89 887 women. The Lancet **1996**;347(8999):431-6.
135. Braüner CM, Overvad K, Tjønneland A, Attermann J. Induced abortion and breast cancer among parous women: a Danish cohort study. Acta obstetricia et gynecologica Scandinavica **2013**;92(6):700-5.
136. Cancer CGoHFiB. Menarche, menopause, and breast cancer risk: individual participant meta-analysis, including 118 964 women with breast cancer from 117 epidemiological studies. The lancet oncology **2012**;13(11):1141-51.
137. Wu Y, Zhang D, Kang S. Physical activity and risk of breast cancer: a meta-analysis of prospective studies. Breast cancer research and treatment **2013**;137:869-82.
138. Steindorf K, Ritte R, Eomois PP, Lukanova A, Tjonneland A, Johnsen NF*, et al.* Physical activity and risk of breast cancer overall and by hormone receptor status: the European prospective investigation into cancer and nutrition. International journal of cancer **2013**;132(7):1667-78.
139. Gaudet MM, Gapstur SM, Sun J, Diver WR, Hannan LM, Thun MJ. Active smoking and breast cancer risk: original cohort data and meta-analysis. Journal of the National Cancer Institute **2013**;105(8):515-25.
140. Pirie K, Beral V, Peto R, Roddam A, Reeves G, Green J*, et al.* Passive smoking and breast cancer in never smokers: prospective study and meta-analysis. International journal of epidemiology **2008**;37(5):1069-79.
141. Kungu A, Hamajima N, Hirose K. Alcohol, tobacco and breast cancer--collaborative reanalysis of individual data from 53 epidemiological studies, including 58,515 women with breast cancer and 95,067 women without the disease. **2002**.
142. Jung S, Wang M, Anderson K, Baglietto L, Bergkvist L, Bernstein L*, et al.* Alcohol consumption and breast cancer risk by estrogen receptor status: in a pooled analysis of 20 studies. International journal of epidemiology **2016**;45(3):916-28.
143. Missmer SA, Smith-Warner SA, Spiegelman D, Yaun S-S, Adami H-O, Beeson WL*, et al.* Meat and dairy food consumption and breast cancer: a pooled analysis of cohort studies. International journal of epidemiology **2002**;31(1):78-85.
144. Dong J-Y, Zhang L, He K, Qin L-Q. Dairy consumption and risk of breast cancer: a meta-analysis of prospective cohort studies. Breast cancer research and treatment **2011**;127:23-31.
145. Aune D, Chan D, Vieira A, Rosenblatt DN, Vieira R, Greenwood D*, et al.* Fruits, vegetables and breast cancer risk: a systematic review and meta-analysis of prospective studies. Breast cancer research and treatment **2012**;134:479-93.
146. Trock BJ, Hilakivi-Clarke L, Clarke R. Meta-analysis of soy intake and breast cancer risk. Journal of the National Cancer Institute **2006**;98(7):459-71.

147. Reeves GK, Pirie K, Green J, Bull D, Beral V, Collaborators MWS. Comparison of the effects of genetic and environmental risk factors on in situ and invasive ductal breast cancer. International journal of cancer **2012**;131(4):930-7.
148. Cheraghi Z, Poorolajal J, Hashem T, Esmailnasab N, Doosti Irani A. Effect of body mass index on breast cancer during premenopausal and postmenopausal periods: a meta-analysis. PloS one **2012**;7(12):e51446.
149. Beral V, Reeves G, Bull D, Green J, Collaborators MWS. Breast cancer risk in relation to the interval between menopause and starting hormone therapy. Journal of the National Cancer Institute **2011**;103(4):296-305.
